# Modern Trends in Polymerization-Induced Self-Assembly

**DOI:** 10.3390/polym16101408

**Published:** 2024-05-15

**Authors:** Natalia S. Serkhacheva, Nickolay I. Prokopov, Evgenii A. Lysenko, Elena Yu. Kozhunova, Elena V. Chernikova

**Affiliations:** 1 Lomonosov Institute of Fine Chemical Technologies, MIREA—Russian Technological University, pr. Vernadskogo, 86, 119571 Moscow, Russia; prokopov@mirea.ru; 2 Faculty of Chemistry, Lomonosov Moscow State University, Leninskie Gory 1, bld. 3, 119991 Moscow, Russia; evglys1970@gmail.com (E.A.L.); kozhunova@polly.phys.msu.ru (E.Y.K.); 3 Faculty of Physics, Lomonosov Moscow State University, Leninskie Gory 1, bld. 2, 119991 Moscow, Russia

**Keywords:** polymerization-induced self-assembly, AB- and ABC-block copolymers, gradient copolymers, morphological transitions, emulsion polymerization, dispersion polymerization, stimuli-responsive polymers, aminoxyl-mediated polymerization, reversible addition–fragmentation chain transfer polymerization, atom transfer radical polymerization, ring-opening polymerization, anionic polymerization, degenerative chain transfer, polymerization-induced thermal self-assembly, polymerization-induced cooperative assembly, polymerization-induced electrostatic self-assembly, polymerization-induced hierarchical self-assembly, polymerization-induced particle self-assembly

## Abstract

Polymerization-induced self-assembly (PISA) is a powerful and versatile technique for producing colloidal dispersions of block copolymer particles with desired morphologies. Currently, PISA can be carried out in various media, over a wide range of temperatures, and using different mechanisms. This method enables the production of biodegradable objects and particles with various functionalities and stimuli sensitivity. Consequently, PISA offers a broad spectrum of potential commercial applications. The aim of this review is to provide an overview of the current state of rational synthesis of block copolymer particles with diverse morphologies using various PISA techniques and mechanisms. The discussion begins with an examination of the main thermodynamic, kinetic, and structural aspects of block copolymer micellization, followed by an exploration of the key principles of PISA in the formation of gradient and block copolymers. The review also delves into the main mechanisms of PISA implementation and the principles governing particle morphology. Finally, the potential future developments in PISA are considered.

## 1. Introduction

Block copolymers consist of at least two types of units, A and B, organized in a block fashion, i.e., AAAA…-…BBBBB. In most cases, due to the thermodynamic incompatibility of the blocks, these polymers exhibit a strong tendency for block segregation in the solid state or in solutions [1,2,3,4,5,6,7,8]. In the solid state, the thermodynamic incompatibility of blocks leads to their micro-segregation. When the A-block is longer, the structure of the solid manifests as fragments of the B-phase (spherical or cylindrical shape) dispersed in an A-matrix. Conversely, when the B-block is longer, the scenario is reversed, with fragments of the A-phase dispersed in a B-matrix. When the lengths of both blocks are comparable, a lamellar morphology is typically observed [1,2,8]. In non-selective solvents (i.e., solvents with approximately similar thermodynamic affinity towards both blocks), the solvation of blocks by solvent molecules masks their incompatibility, resulting in no segregation or self-assembly of block copolymers. Instead, their macromolecules adopt a conformation resembling a random coil [1,2]. In selective solvents (i.e., solvents that are good solvents for one of the blocks, such as the A-block, while being very poor or even non-solvents for the other block, e.g., the B-block), macromolecules are organized into core-corona structures known as micelles [1,3,5,6,7]. Micelles consist of a collapsed insoluble core formed by B-blocks, surrounded by a solvated lyophilizing corona composed of units from the A-block.

Block copolymerization, when performed in a selective solvent for the growth of a block, may result in the self-assembly of the block copolymer into micelles, followed by the formation of polymer particles with different morphologies. This process, known as polymerization-induced self-assembly (PISA), has been developed recently and has attracted the attention of numerous research teams due to its ability to produce stable colloidal dispersions of block copolymer particles with tunable morphologies without the need for any surfactants or steric stabilizers [9]. The progress in the synthesis of well-defined block copolymers organized into particles with diverse morphologies has been discussed extensively in numerous research articles and reviews [10,11,12,13,14,15]. However, many of these reviews focus on a specific aspect of the PISA process, such as the specific mechanism of implementation [10,13], progress in dispersion or emulsion-based PISA [12], potential applications of PISA [16], or control of particle morphology through PISA [9,15].

In the present review, we have tried to bring together the array of information dealt with by PISA and to provide an overview of the current state of rational synthesis of polymeric particles with diverse morphologies using various PISA techniques and mechanisms. We discuss the main thermodynamic, kinetic, and structural aspects of AB- and ABC-block copolymer micellization, which determine the type of particle morphology formed during PISA, and the main mechanisms of PISA implementation. This review also delves into the main switchable tools provoking self-assembly during polymerization, i.e., temperature-induced phase separation or crystallization of blocks, parallel synthesis of the homopolymer, strong electrostatic interactions between blocks, and surface interactions of growing copolymers with colloid particles. Finally, the computer simulation studies and potential future developments in PISA are considered. The main feature of this review is its focus on the correlation between the chemical composition of growing macromolecules, synthetic mechanisms, and resulting particle morphologies.

## 2. The Basic Principles of Block Copolymer Self-Assembly

### 2.1. Thermodynamics, Kinetics, and Mechanism of AB-Diblock Copolymer Micellization

The process of transformation of block copolymer macromolecules from a single coil in solution (M) into part of a micelle state (M)*_mic_* with micelle aggregation number *N* can be represented as $M⇌1/N{\left(M\right)}_{mic}$. Then, for equilibrium, the micellization constant (*K_mic_*) can be written as follows:

(1) $$K_{mic}=\frac{{\left[{\left(M\right)}_{mic}\right]}^{1/N}}{\left[M\right]}$$

When *N* >> 1, Equation (1) simplifies to the following:

(2) $$K_{mic}=\frac{1}{\left[M\right]}$$

At a copolymer concentration above the critical micelle concentration (CMC), one can assume [M] ≅ CMC. Therefore,

(3) $$\Delta G_{mic}^{0}=-RTln\left(K_{mic}\right)≅RTln\;CMC\;\mathrm{and}\;\Delta H_{mic}^{0}=-RT^{2}\frac{dln\;CMC}{dT}\;,$$

where Δ*G*^0^*_mic_* and Δ*H*^0^*_mic_* are standard Gibbs energy and standard enthalpy of micellization [1,2]. Thus, the CMC represents itself as a characteristic value that completely describes the micellization thermodynamics of block copolymers [1,17,18].

The existence of CMC in block copolymer’s solutions has been demonstrated experimentally in a number of publications [17,18,19,20,21,22,23]. Usually, CMC values have the order of 10^−5^–10^−8^ M, i.e., 3–5 orders less than CMC values for regular low-molecular-weight surfactants. As a rule, micellization of block copolymers becomes permissible when the length of the insoluble block exceeds 4–6 units, depending strongly on the chemical nature of the solvent, the chemical nature, and the length of the insoluble B-block, while the dependence on the chemical nature and the length of the soluble A-block is less prominent [17,18,20,21,24]. For example, for the aqueous solution of block copolymers of polystyrene and sodium polyacrylate PS-*b*-PANa, an increase in the length of PS block from 6 to 110 units decreases the CMC by 320 times, while an increase in the length of PANa block from 300 to 1400 units enhances the CMC by less than twice [17].

The study of the micellization kinetics of block copolymers upon instantaneously “switching on” the selectivity of the solvent by a temperature change has revealed a two-step mechanism of micelle formation [25,26]. During the first step, there is swift, spontaneous nucleation of block copolymer macromolecules into small pre-micelles. All macromolecules initiate the nucleation process almost simultaneously, thereby generating a narrow size distribution of pre-micelles. The nucleation process halts when the macromolecule concentration falls below the CMC.

In the second step, a slow relaxation process of reorganization of pre-micelles into equilibrium structures occurs. This process involves the enlargement of certain pre-micelle aggregates while their overall quantity gradually decreases. The reorganization process is facilitated by either the transfer of a single macromolecule from one pre-micelle to another through the solvent or by the fusion of pre-micelles into larger aggregates with subsequent reorganization [25,26,27].

Both mechanisms are connected with activation barriers arising from the transfer of insoluble blocks through the bulk of non-solvent and the incompatible corona of the second block. Very often, these barriers are so high that the process freezes and an equilibrium state is not achieved. Only block copolymers with relatively short (approximately 10–30 units) and “soft” insoluble blocks with a glass transition temperature far below that used in experiments could spontaneously form equilibrium micelles upon direct dispersion of polymer material in a selective solvent [20,21,23,24].

Two approaches are applied to promote micelle formation. The first one involves increasing the kinetic energy of block copolymer macromolecules to enable them to overcome the activation barrier. In this case, the process of direct dissolution of block copolymers is carried out at elevated temperatures very close to the boiling point of the solvent under vigorous stirring until micelle aggregates of constant dimensions are formed. This approach allows for the preparation of stable micelle solutions if the length of the insoluble block does not exceed approximately 100 units, while the length of the soluble block is much higher [1,28].

The second approach involves initially decreasing the activation barrier by temporarily reducing the selectivity of the solvent. In this method, the block copolymer is first dissolved in a non-selective solvent, followed by a gradual substitution of the non-selective solvent with a selective one, typically using dialysis techniques. This approach is considered to be the most versatile and allows for the preparation of micelle solutions with reproducible characteristics for block copolymers of varying lengths of their soluble and insoluble blocks. However, the extent to which the micelles obtained using this method approach equilibrium remains a topic of debate and has not yet yielded a definitive answer [27,29,30].

PISA constitutes an alternative approach for kinetic control for block copolymer self-assembly. At the initial stages of the reaction, when the length of the growing solvophobic block is small, forming micelles fits the equilibrium conditions. Upon further proceeding with the reaction, the kinetic lability of the solvophobic block becomes restricted, though not completely, due to the plasticization of the core by monomer molecules. Therefore, in a PISA process, both thermodynamic and kinetic control of particle morphologies are possible.

### 2.2. Structure and Morphology of AB-Diblock Copolymer Micelles

**Figure 1:**
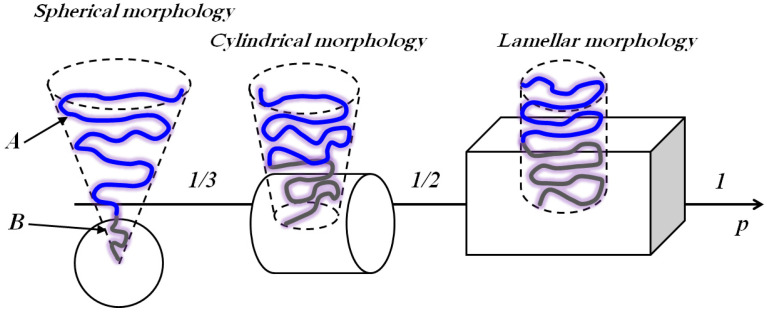
Possible morphologies of AB-block copolymer micelles and their relationship with packing parameter *ρ* [3].

In selective solvents, micelles of diblock copolymers may exist in one of three characteristic morphologies: spherical, cylindrical (worm-like), or lamellar (Figure 1). The manifestation of a certain morphological type is defined, like for low-molecular-weight surfactants, by the value of the packing parameter *ρ*. For polymer coils, such parameters may be defined as follows:

(4) $$\rho \sim \frac{\upsilon_{B}}{\upsilon_{A}}$$

where υ_B_ and υ_A_ represent spatial volumes occupied by insoluble (B) and soluble (A) blocks, respectively. As a rule, spherical micelles form when *p* ≤ 1/3, cylindrical micelles at 1/3 < *p* ≤ 1/2, and lamellar structures (bilayers and vesicles) are realized within the interval 1/2 < *p* ≤ 1 [3,31]. When *ρ >* 1, the formation of so-called “large compound micelles” is possible. In this case, the block copolymer particle represents itself as a cocoon forming small reverse micelles with the core of soluble blocks and corona from insoluble blocks. Such cocoons are wrapped by a thin shell of single macromolecules with insoluble blocks inward, anchoring insoluble corona of reversed micelles and soluble blocks outward, and stabilizing the whole structure via solvation by solvent molecules [32].

The main thermodynamic reason for such transformations lies in the interplay of free Gibb’s energies of corona (*F*_corona_), core (*F*_core_), and surface energy at the core–corona interface (*F*_sur_). Therefore, the total free energy of the micelle (*F*) may be represented as: *F* = *F*_corona_ + *F*_sur_ + *F*_core_ [3,33,34,35]. When υ_B_ << υ_A_, *F*_corona_ dominates. This term is determined by the strong repulsion of swollen A-blocks in the corona. To alleviate such repulsion and minimize *F*_corona_, spherical morphology seems to be the most suitable, since it provides maximum space for A-block lodging within the micelle. In such micelles, the width of the corona is much larger than the radius of the core; therefore, they get the name “star-like” micelles [1,22].

When ρ is increasing, and hence the core-corona interface is growing too, the term *F*_sur_ starts to prevail within the sum. To diminish specific surface energy per macromolecule, spherical micelles have to grow, forming so-called “crew-cut” spherical micelles, where the radius of the core is much larger than the width of the corona [1,22].

The growth of the core radius within the sphere is accompanied by the elongation of B-blocks and their much greater deviation from the unperturbed coil conformation, thus causing the growth of *F*_core_. When ρ exceeds 1/3, the penalty of B-block elongation becomes unbearable, and micelles turn to non-spherical morphologies, where both *F*_sur_ and *F*_core_ may simultaneously decrease, while B-block may adopt a coil conformation again [3,32,35].

Both υ_B_ and υ_A_ are proportional to block lengths, but such proportionality is quite different for both blocks. Since A-block is highly swollen, small changes in its length cause significant changes in υ_A_. Since the B-block has significantly collapsed, significant changes in its length cause small changes in υ_B_. Therefore, theoretical recalculations of the packing parameter into the block length ratio P_B_/P_A_ (P_B_ and P_A_ are degrees of polymerization of corresponding blocks) predicts the following criteria for the stability of certain morphologies: When P_B_/P_A_ ≤ 1, star-like spherical morphologies are thermodynamically stable, while non-spherical (cylinder and lamellar) morphologies become possible, when P_B_/P_A_ >> 1. If P_B_/P_A_ > 1, both crew-cut spherical and non-spherical morphologies are possible, depending on the chemical nature of both blocks and the selectivity of the solvent [32,34,35,36].

Theoretical predictions are in good agreement with experimental observations [26,32,37]. For example, for block copolymers of polystyrene (PS) and polyacrylic acid (PAA) in aqueous media, the formation of spherical micelles was observed for the PS_500_-b-PAA_50_ copolymer (subscript indexes designate polymerization degrees of corresponding blocks) with P_PS_/P_PAA_ = 10, while cylindrical (worm-like) micelles were found for PS_180_-b-PAA_15_ (P_PS_/P_PAA_ = 12). In the case of the PS_410_-b-PAA_20_ copolymer (P_PS_/P_PAA_ = 20), small vesicles with a lamellar structure were formed [32].

Because of the swelling of soluble blocks in micelle corona, their effective volume υ_A_ is essentially sensitive to the thermodynamic quality of the solvent. Small changes in solvent quality may trigger significant morphological reorganization, especially in the field of non-spherical morphologies [33,34,37,38]. Thus, the consequential addition of HCl into aqueous micelle solutions of PS_410_-b-PAA_13_ suppresses the PAA dissociation and deteriorates the solvent quality towards PAA-block, thus diminishing its υ_A_. As a result, a morphological transition from spherical to cylindrical shape is observed, while the addition of HCl stimulates the reverse reorganization. The addition of NaCl into aqueous dispersions of PS_410_-b-PAA_25_ stimulates a gradual morphological transition in the following order: spheres → cylinders → vesicles, while the addition of CaCl_2_ triggers the direct transformation of spherical morphology into a lamellar one, skipping the formation of a cylindrical shape [37].

In highly selective solvents, the micellar core represents itself as a “drop” of almost pure B-block with a density close to that of B-homopolymer in a solid state [5,28,29,39,40]. For star-like micelles, the conformation of B-blocks in a core is close to the unperturbed conformation of a random coil [28,40]. The core may exist in a glassy state (if the glass transition temperature of B-block exceeds the experimental one) or in a highly elastic state (if the glass transition temperature of B-block lies far below the experimental one). In the first case, micelles are kinetically frozen and cannot change their morphology or aggregation number. In the second case, the formation of labile micelles able to undergo structural reorganizations as a response to external stimuli becomes possible [41].

Like the micelles of regular surfactants, block copolymer micelles are able to solubilize low-molecular-weight substances. The most investigated are processes of solubilization of non-polar substances, both liquid (benzene, chloroform, chlorobenzene, cyclohexane, etc.) and solid (pyrene, phenanthrene, triclosan, thiocyanomethylthiobenzotiazole, etc.), into the hydrophobic core of block copolymer micelles with nonpolar B-core and polar A-corona in aqueous media [42,43,44,45,46,47]. It was shown that the values of the distribution coefficients of solubilized substances between aqueous and B-block phases as well as the loading capacities of block copolymer micelles were comparable with those of micelles of regular low-molecular-weight surfactants. Nevertheless, the kinetics of solubilization and re-solubilization for block copolymers were much slower, especially for micelles with glassy cores [45].

Solubilization of B-homopolymer is also possible into the micelle core if its length does not exceed the length of B-block and the loading path goes through joint dissolution of block copolymer and homopolymer in a nonselective solvent with subsequent dialysis of the obtained mixture against the selective one [1,34,48]. Solubilization plasticizes the core and alleviates the elastic strain of B-blocks, thus diminishing *F*_core_ and stabilizing the spherical morphologies of copolymer micelles [48].

Micellization of triblock copolymers with “split” A- or B-block, e.g., ABA or BAB in general, obeys the same thermodynamic, kinetic, and morphological features as were described above for AB-diblock macromolecules [1]. The ABA-triblock copolymers usually have higher values of CMC and lower aggregation numbers due to better shielding of insoluble B-block by two soluble ones, while their spherical morphology is more stable in comparison with AB-diblock copolymers with the same overall numbers of A- and B-units. For BAB-triblock copolymers, the formation of loops of A-blocks in corona is typical, as is gel formation via association of the outward B-blocks upon increasing the overall block copolymer concentration [2].

For A-block-B/A copolymers, the core-forming block (B/A) of which represents itself as a random or gradient copolymer from B- and A-units, the formation of labile micelles capable of changing their aggregation number and dimensions upon change in environmental conditions was observed when the content of A-units in A/B-block approaches ca. 50 mol. %. At higher levels of A-units, such block copolymers lose their micellization ability [49,50,51,52,53,54,55].

In the inverted case of B-block-A/B copolymers with insoluble B-units distributed randomly within the corona-forming A/B-block, the formation of micelles with a contracted corona and an enlarged micelle core was observed in dilute solutions when the content of B-units in the A/B-block exceeded ca. 50 mol. %. The contraction of the corona was caused by the statistical association of insoluble B-units in the amphiphilic corona [56,57]. At higher micelle concentrations, inter-corona association of insoluble B-units leads to the formation of micellar gels [58,59,60,61].

### 2.3. Structure and Morphology of ABC-Triblock Copolymer Micelles

Being dispersed in a selective solvent, ABC-triblock copolymers form micelles with a two-section B/C-core, surrounded by lyophilic A-corona [62,63,64]. Two variants of B/C-core morphology are possible. The first variant is known as “onion-type”. In this case, a single core of one block (e.g., B) is completely immersed into a single core of another one (e.g., C) [64,65,66,67]. In a second variant, the formation of a so-called “raspberry-type” core is observed, and multiple “drops” of one block (e.g., C) are partially embedded into the single continuous core of another lyophobic block (e.g., B) [64,65,68].

The morphology of the two-section core is determined by the degree of thermodynamic incompatibility of both blocks as well as by the ratio of their lengths. If both blocks are moderately incompatible and comparable in length, then onion-type micelles do form. For example, such micelles were observed for triblock copolymers from poly(2-ethylhexyl)acrylate (PEA), poly(methyl methacrylate) (PMMA) and PAA, PEA_31_-b-PMMA_56_-b-PAA_195_ [66] and triblock copolymer from PS, poly(2-vinylpyridine) (P2VP), and polyethylene oxide (PEO), PS_190_-b-P2VP_130_-b-PEO_190_ (pH > 5) in aqueous dispersions [67].

In the case of high B- and C-block incompatibility (e.g., one block is hydrocarbon, while the second one is fluorocarbon) or a significant difference in block lengths, raspberry-type micelles do so. ABC-copolymer from poly(pentafluorophenyl 4-vinylbenzoate)_10_-b-PS_50_-b-poly(4-methyl-4-(4-vinylbenzyl)morpholinium chloride)_70_ in aqueous media may serve as a typical example of such structures [68].

Micelles with a two-section hydrophobic core may manifest a synergetic effect upon the solubilization of low-molecular-weight organic substances from aqueous solutions [66], as well as the ability for selective solubilization of two different nonpolar substances (drugs, dyes, etc.) into different sections of the core [69]. Such property opens perspectives for the practical application of such micelles for concurrent delivery of several different drugs within the frame of one nanocontainer [70]. In some cases, such two-section cores may also serve as nanoreactors for the synthesis and stabilization of metal nanoparticles [67].

In this case, two lyophilic A- and C-blocks are presented in corona. The corona structure is defined by the sequence of connections between blocks and their thermodynamic compatibility. For block copolymers, BAC micelles with two-layered “onion-type” coronas with spatially separated A- and C-blocks do form. A-blocks are connected with insoluble B-block and located in the inner part of the corona, while C-blocks form the corona’s periphery. In this case, radial segregation of soluble blocks is observed [63,71,72,73].

When insoluble B-block is intermediate, i.e., we have the sequence ABC, two variants are possible. If blocks are compatible, the formation of micelles with joint coronas with intermixed A- and C-blocks is observed. If A- and C-blocks are incompatible, segregation of C- and A-blocks in corona has its place. The ultimate case of such segregation manifests itself in the formation of so-called Janus micelles. In Janus micelles, A- and C-blocks form two semi-spheres, which surround the core from two different sides. In this case, the lateral segregation of soluble blocks occurred [31,74,75].

If one or both soluble blocks are able to dissociate ionically, different micelle morphologies of block copolymers may be monitored, depending on the block ionization degree [76,77,78,79]. For example, for block copolymers P2VP_260_-b-PMMA_50_-b-PAA_320_ in highly acidic aqueous media (pH < 2), small spherical micelles from ca. 10 macromolecules are formed. Micelles consist of B core and mixed PAA/P2VPH^+^ corona. At higher pH values, micelles precipitate due to progressive intermicellar aggregation, caused by the formation of salt bonds between oppositely charged P2VPH^+^ and PAA^−^ units. When pH > 5.8, P2VP units become deionized and, hence, lose their solubility in water. In this case, the formation of Janys micelles with a PMMA core surrounded by two hemispheres from P2VP and PAA blocks is observed. Micelles are subjected to further aggregation into large, loose structures through their P2VP semi-spheres in order to avoid contact between P2VP blocks and their aqueous surroundings [77].

An amazing feature of ABC-block copolymers is their ability to form myriad morphologies ranging from “simple” spheres, cylinders, and lamellae up to complex multi-segregated nanoparticles of intriguing shapes [6,7,8,38,48,61,69,78,79]. The complex morphologies have been given unusual names that reflect the creative thinking of the researchers who first discovered them. For example, they are referred to as “flower-like” [80], “jellyfish-like” [81], and so on. The “updates” of these names in modern literature have not ignored the more traditional cylindrical and lamellar morphologies, which are now known as nano-rods [9], worms [82,83,84,85], and vesicles (polymersomes) [82,83,84,85]. The tendency for unusual morphologies grows with an increase in the chemical difference of the constituent blocks and the asymmetry in their lengths. Strong interactions between units within the same block (e.g., crystallization) or between units of different blocks (e.g., formation of interpolymer complexes) significantly promote the formation of irregular morphologies. The reason for such diversity lies in the effect of the third block. For AB-diblock copolymers, “simple” morphology provides good protection for AB-interfaces against their contacts with the solvent. However, in the case of ABC-triblock copolymers with two insoluble and incompatible blocks, there will be three such surfaces: AB-, AC-, and BC-. In general, no one “simple” morphology could provide enough protection for each of these three. As a result, if initially a polymer nanoparticle adopts one of three main morphologies (sphere, worm, or polymersome), it will still have “sticky” surface patches with abundant free energy. Secondary interparticle aggregation of such patches will provide the formation of morphologies of high order. The elaboration of a robust theory of morphological transitions in ABC-block copolymers is a fascinating challenge in this field.

Concluding this section, we can state that due to the large relaxation times of insoluble blocks in selective solvents, the preparation of micellar solutions, even in the case of AB-diblock copolymers, represents a rather continuous and often laborious multi-step procedure in which the equilibrium state is often not achievable. Therefore, the PISA approach may be a strong alternative to the traditional synthesis and micellization routine. The main advantage of PISA lies in its ability to substitute a long set of sequential steps (synthesis, isolation, purification, dissolution, and micellization) with a simple one-pot procedure.

For ABC-triblock copolymers, the advantages of PISA are even more prominent. It is very difficult to find a non-selective solvent for three blocks with essentially different chemical natures in their units. Hence, the facilities for the synthesis of ABC-triblock copolymers of the desired composition were greatly restricted before the appearance of PISA. As will be seen below, the PISA approach enables practically any combination of blocks with any block length ratio. As a result, PISA greatly expands the assortment of available monomer combinations and, hence, the assortment of available block copolymer morphologies. At this stage of the development of the field, it is very important to find correlations between the chemical nature of monomer units, the mechanism of copolymer synthesis, and the resultant particle morphology. This important task will be the focus of further discussion.

## 3. General Idea of Polymerization-Induced Self-Assembly

Polymerization-induced self-assembly (PISA) is based on the well-known ability of amphipathic macromolecules to form nano- or microstructures of various morphologies under the action of the proper stimulus. Amphipathic macromolecules may have various monomer unit sequence distributions and include random, alternate, gradient, and block copolymers. However, only block and gradient copolymers have pronounced diphilic structure opposite to random and particularly to alternating copolymers, which determine their ability to self-assemble in a selective solvent and to participate in PISA.

**Scheme 1:**
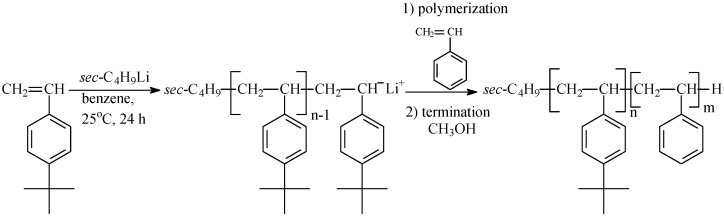
Synthesis of a steric stabilizer based on poly(*t*-butyl styrene)-*b*-polystyrene [86].

**Scheme 2:**
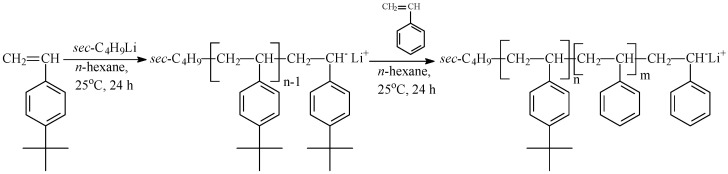
Dispersion anionic polymerization of styrene initiated by poly(*t*-butyl styryl)lithium [86].

The keystone idea of PISA was developed in the late 1990s during the study of dispersion anionic polymerization of styrene [86,87,88]. Kim et al. [86] have discovered that poly(*t*-butyl styrene)-*b*-polystyrene pre-formed (Scheme 1) or formed in situ (Scheme 2) can act as a steric stabilizer in anionic dispersion polymerization of styrene in *n*-hexane and provide the formation of stable dispersions with a relatively narrow particle size distribution. In both cases, the molecular weight (MW) of poly(*t*-butyl styrene) plays a crucial role in the stabilizing ability of block copolymers.

Stable dispersions with an average particle diameter of 60–100 nm were formed even when the mixture of *sec*-C_4_H_9_Li and poly(*t*-butyl styryl)lithium was added to the styrene polymerization in *n*-hexane. The simultaneous generation of polystyrene and block copolymers of poly(*t*-butyl styrene)-*b*-polystyrene also resulted in the formation of stable dispersions. A detailed study of the mechanism of particle formation was performed in [87]. Dispersion polymerization of styrene initiated by *sec*-C_4_H_9_Li was conducted in *n*-hexane in the presence of polystyrene-*b*-polybutadiene as a stabilizer. The particle nucleation did not occur in the micelles of the block copolymer stabilizer or in monomer droplets (as the monomer was soluble in *n*-hexane). Instead of this, the polystyrene chains formed via the anionic polymerization mechanism grew to critical length, which was limited by their solubility in the *n*-hexane/styrene medium. The loss of solubility caused chain aggregation and adsorption of stabilizer macromolecules, followed by the formation of primary particles and their further growth due to styrene polymerization. A similar mechanism should take place when poly(*t*-butyl styryl)lithium initiates the anionic polymerization of styrene [86], with the exception that poly(*t*-butyl styrene) is a stabilizer and initiator simultaneously. The same approach of using the double functional living polymer was applied in [88] for the synthesis of highly cross-linked 1,4-divinylbenzene microgels with a shell of poly(*t*-butyl styrene). Further investigations of the formation of polyisoprene-*b*-polystyrene through living anionic dispersion polymerization have confirmed that block copolymers formed underwent self-assembling during the synthesis [89]. This process was called Polymerization-Reaction-Induced Molecular Self-Assembling (PRIMSA).

Thus, the combination of initiating and stabilizing abilities in one macromolecule gave rise to the exploitation of a new approach to the synthesis of macromolecules able to self-assemble in the process of polymerization. However, living anionic polymerization has numerous restrictions on the experimental settings, including the high purity of reagents, the absence of moisture, a narrow range of monomers, etc. For this reason, its attractiveness for further developing PRIMSA was relatively low.

Radical polymerization in the dispersed media, on the contrary, is frequently used for the production of polymer dispersions that are highly demanded in many areas, including adhesives, surface coatings, biomedical applications, etc. [90,91,92,93,94,95,96,97,98,99,100]. Depending on the solubility of the monomer in the dispersion medium and the mechanism of particle nucleation, different sizes of the polymer particles can be achieved (from dozens of nm to dozens of μm) [101,102,103,104]. Meanwhile, the capacity of classical radical polymerization in dispersed media is restricted to the synthesis of homopolymers and random copolymers. The potential of radical polymerization was reconceived due to the evolution of reversible deactivation radical polymerization (RDRP) that came into the spotlight in the late 1990s [102,103,104,105]. It is worth reminding ourselves that RDRP has features similar to living anionic polymerization, which include the linear rise of number-average MW with the progress of monomer conversion, the narrow MWD of the polymer, and the ability of the polymer to undergo chain extension after the addition of a new or the same monomer. The principal differences between RDRP and living polymerization are the following: In RDRP, the macromolecules grow stepwise due to periodic activation–deactivation of propagating radicals, while in living polymerization no deactivation reactions occur; irreversible termination of propagating species occurs only during RDRP.

**Scheme 3:**
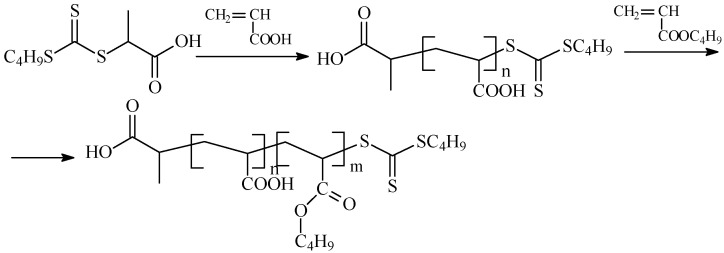
RAFT-mediated emulsion polymerization of *n*-butyl acrylate in the presence of oligomeric polyacrylic acid terminated by trithiocarbonate [111].

The first attempts to apply RDRP to the emulsion or dispersion process ended in failure: stability of the polymer dispersions was usually low, particle size distribution was broad, and control of MWD was poor [106,107,108,109,110]. These problems came from the poorly controlled nucleation of particles. In contrast, RDRP in solution or in bulk has provided high control over MWD and chain architecture. Finally, the advances in controlled synthesis of block and gradient copolymers by RDRP pushed the researchers closer to PRIMSA through radical mechanisms [111,112,113,114,115,116,117,118,119,120,121]. The first example of PRIMSA in radical polymerization was given in [111]. This work involves the synthesis of oligomeric acrylic acid through reversible addition–fragmentation chain transfer (RAFT) polymerization in aqueous media, followed by block copolymerization with butyl acrylate added under controlled feed in the same media (Scheme 3).

**Scheme 4:**
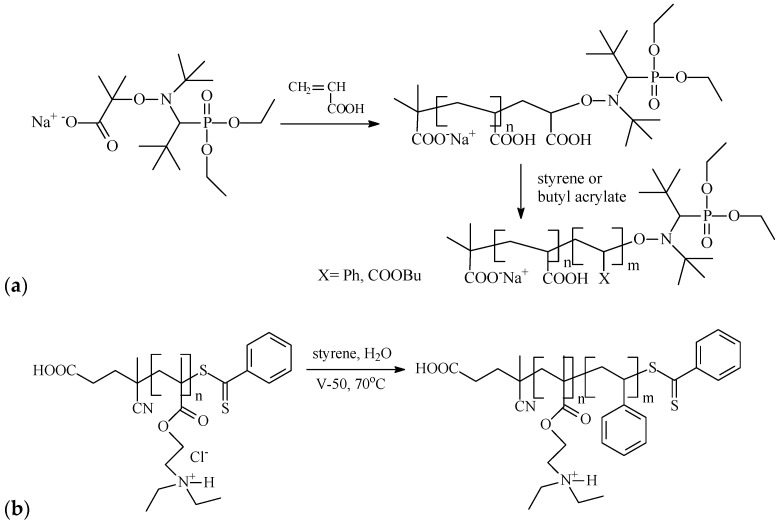
Aminoxyl-mediated emulsion polymerization of styrene and *n*-butyl acrylate in a single batch step (**a**) and batch emulsion polymerization of styrene stabilized by a macro-RAFT agent (**b**) [122,123].

A few years later, in a series of publications, the Charleux group developed one-step batch emulsion polymerization using hydrophilic macroinitiators (aminoxyl-mediated radical polymerization) (Scheme 4a) [122] or macro-RAFT agents (Scheme 4b) [123]. Latex particles formed in both cases have a spherical form typical for emulsion polymerization.

The direct single-step synthesis of vesicles through aminoxyl-mediated emulsion polymerization in 2009 by the Charleux group has become a breakthrough for producing particles with the desired morphology [124]. The term “polymerization-induced” formation of block copolymer dispersions was introduced first. Finally, Pan et al. [9] have introduced the term PISA, which is widely used now for heterogeneous RDRP polymerizations associated with macromolecule self-assembly. In contrast to the Charleux group, Pan et al. have described the first dispersion RAFT polymerization, providing the formation of nanorods. Since that time, PISA has been met with great interest due to its ease of implementation and wide possibilities for reaching the desired morphologies of polymer dispersions.

**Figure 2:**
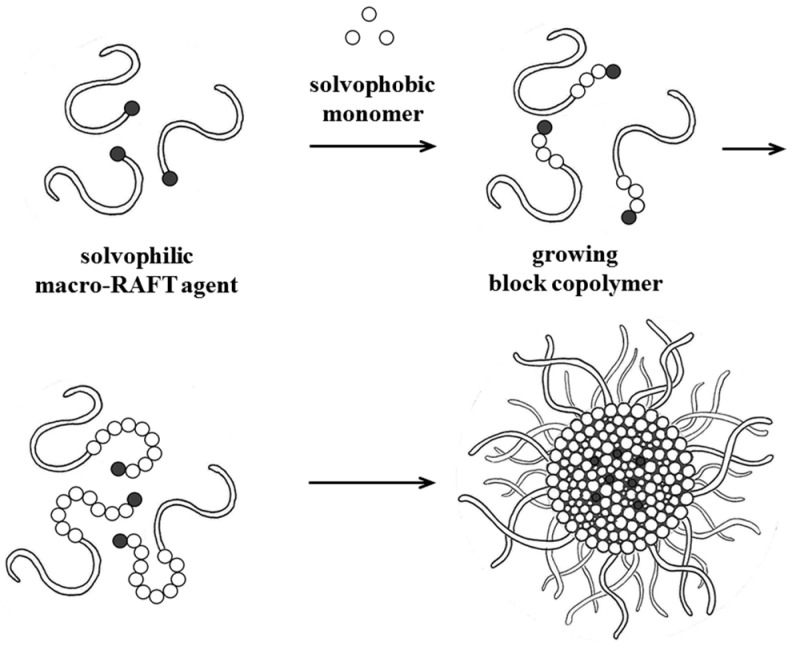
Schematic presentation of the RAFT PISA process [9].

The idea of PISA suggested by Pan et al. [9] is similar to the idea of PRIMSA discussed above [89]. The polymerization is performed in a reaction medium that is selective to the growing block of the forming block copolymer. During PISA, the solvophilic block is produced first, and it acts as a macroinitiator (or macro-RAFT agent) and a stabilizer for the growing solvophobic block (Figure 2). After the length of the solvophobic block reaches a critical value, the phase separation yields the formation of polymer particles. Their morphology depends on the polymerization conditions and may transform in the course of the polymerization due to the change in the ratio of hydrophobic to hydrophilic blocks.

Below, we will briefly discuss the strategies for controlling the synthesis of dispersions of block and gradient copolymers through RDRP.

### 3.1. Block Copolymers

**Figure 3:**
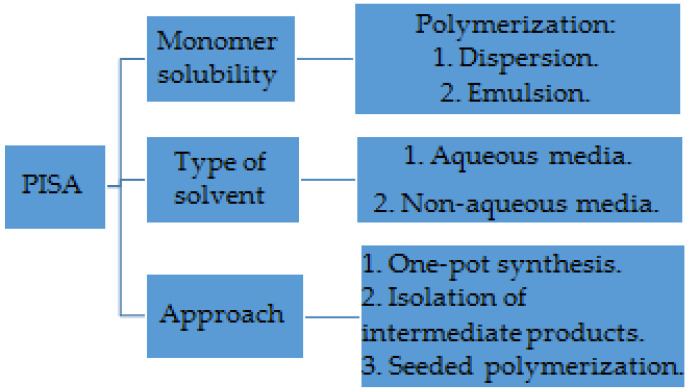
The strategy of PISA implementation.

Block copolymers are the original objectives of PISA. Their ability to self-assemble into the desired morphology becomes possible if block copolymers comprise thermodynamically incompatible blocks of the defined polymerization degree [125]. The strategy of PISA implementation is determined by the chemical nature of the monomers and the desired particle morphology (Figure 3).

To perform PISA, the reaction medium is chosen in a way to be a non-solvent for the core-forming or growing block and a solvent for the shell-forming or stabilizing block. Depending on the monomer solubility in the reaction medium, the choice between dispersion and emulsion polymerization should be made. Dispersion polymerization becomes preferable when both monomers have similar solubility in different solvents opposite to their homopolymers, which should differ in their solubility [126]. The strong differentiation of monomer solvophilicity/solvophobicity is a cause for the choice of emulsion process. Aqueous media are preferred for hydrophilic shell-forming blocks, while organic (non-aqueous) media are used for hydrophobic shell-forming blocks. For AB-diblock copolymer synthesis, a one-pot protocol is preferable, as there is no need for isolation and purification of intermediate shell-forming blocks. It is usually used more for emulsion polymerization than for dispersion polymerization [10,127,128,129]. However, in practice, the isolation of intermediate products (i.e., the initial macro-RAFT agent or macro-aminoxyl) is applied more frequently [130]. The synthesis of dispersions of ABC-triblock copolymers is performed by seeded polymerization [11,131,132,133].

**Scheme 5:**
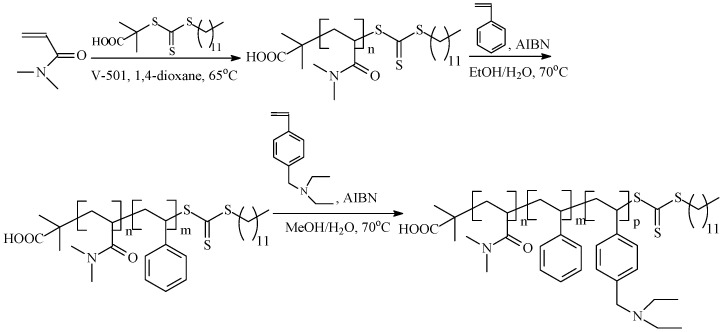
Synthetic route to an ABC-block copolymer dispersion [136].

The morphology of the particles formed during PISA depends on the ratio of the degree of polymerization of shell-forming and core-forming blocks of the AB-diblock copolymer [12,13,14,113,116]. Phase diagrams representing the morphology type as a function of block copolymer composition and degree of polymerization of core-forming blocks have been gained now for numerous systems [15,134,135]. These morphologies typically comprise spheres, worms, and vesicles or their mixtures [82,83,84,85]. For ABA-triblock copolymers, the flower-like morphology becomes possible as well [80]. In the latter case, A-block is a core-forming hydrophobic block, and B-block is a shell-forming hydrophilic block. The number of potential morphologies increases in the case of dispersions of ABC-triblock copolymers. In a typical example, a macro-RAFT agent based on dimethylacrylamide (DMA) was used in the water/ethanol dispersion polymerization of styrene (S), resulting in the formation of AB-diblock copolymer particles (A—shell-forming PDMA block; B—core-forming PS block) (Scheme 5) [136]. The dispersion of seeded particles was subjected to dialysis to replace the solvent mixture, followed by the polymerization of the third monomer, N-(4-vinylbenzyl)-N,N-diethylamine (VEA), and the formation of the dispersion of the ABC-triblock copolymer (A—corona-forming PDMA block; B—shell-forming PS block; C—core-forming PVEA block). The decrease in temperature below the lower critical solution temperature (LCST) of the core-forming PVEA block has resulted in the transformation of spherical particles into unsymmetrical vesicles. The inverse order of the synthesis (PVEA → PS → PDMA) has caused a change in particle morphology directly in the cause of the synthesis due to the increasing polymerization degree of the PDMA block [81]. Vesicles were transformed into tubules; the tubules were converted into jellyfish-like morphology; jellyfish changed into worms; and the latter turned into nanospheres.

### 3.2. Gradient Copolymers

Gradient copolymers as well as block copolymers are able to self-assemble due to solubility differences between distinct segments in the polymer chain [137,138,139,140,141,142]. Hence, gradient copolymers can be considered proper candidates for the PISA process due to a gradual drift in composition along the polymer chain formed during polymerization. Their synthesis can be realized in a one-pot operation by batch copolymerization, if the monomer pair differs strongly by reactivity ratios [143,144,145,146,147], or by continuous or semi-continuous loading of one or both monomers, if the monomers have similar reactivity ratios [148,149]. Self-assembly should occur if homopolymers formed by these monomers are solvophobic and solvophilic concerning the chosen solvent (reaction medium), respectively. Thus, monomer reactivity, monomer feed, the rate of gradual addition of one or both monomers in the reaction, and solvent selectivity are crucial to the self-assembly of gradient copolymers during the synthesis [115,144].

**Scheme 6:**
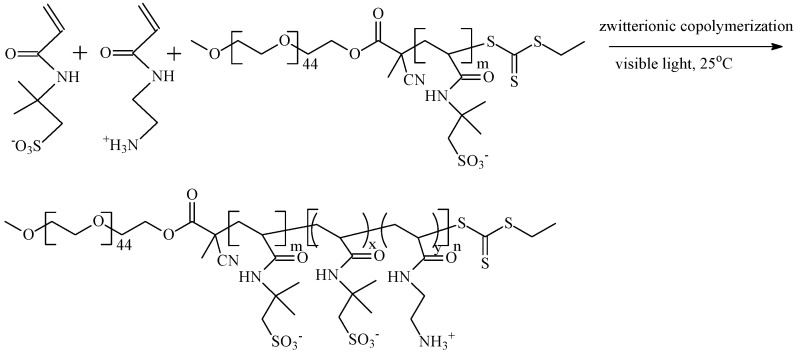
Batch emulsion polymerization of styrene stabilized by a macro-RAFT agent [150].

In the first example of gradient copolymerization-induced self-assembly, the macro-RAFT agent based on poly(ethylene glycol)-based trithiocarbonate (PEG_45_) and 2-acrylamido-2-methylpropanesulfonic acid (AMPS) was prepared first and then used in the copolymerization of AMPS and 2-aminoethylacrylamide hydrochloride (AEAM) of different monomer feeds (Scheme 6) [150]. The gradient copolymer formed in aqueous solutions has both negative and positive charges. The charge stoichiometry and distribution have a marked effect on the copolymer solubility, resulting in self-assembly and the formation of vesicular morphology.

**Scheme 7:**
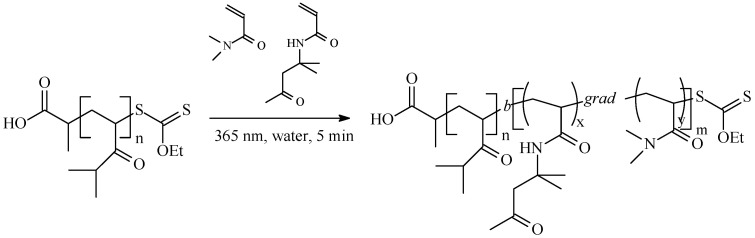
Aqueous photoiniferter PISA of DMA and DAAm [151].

A similar idea was realized in [151] through photoiniferter PISA of DMA and diacetone acrylamide (DAAm) (Scheme 7). Moreover, 2-((ethoxycarbonothioyl)thio)propanoic acid as a photoiniferter was used to polymerize DMA in 1,4-dioxane. After that, an aqueous PISA of DMA (30 mol. %) and DAAm was performed. The spheres, worms, and vesicles formed depended on the copolymer composition and the degree of polymerization.

**Scheme 8:**
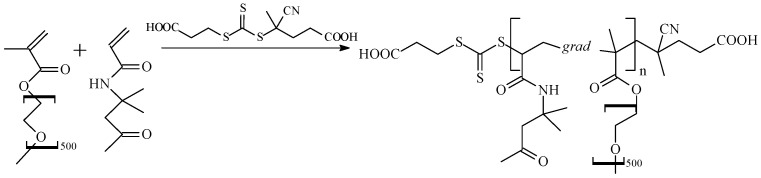
Aqueous PET-RAFT copolymerization of OEGMA and DAAm [152].

**Figure 4:**
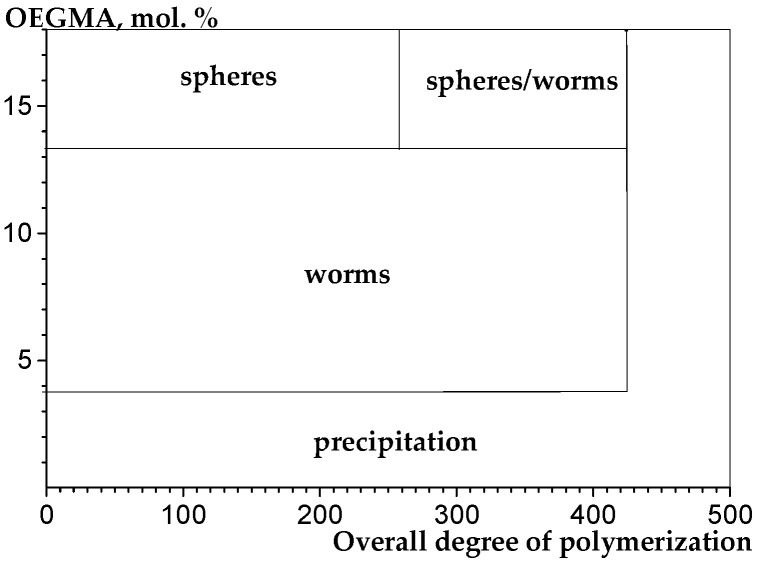
Phase diagram for a series of nanoparticles synthesized using PET-RAFT dispersion copolymerization of OEGMA and DAAm [152].

A one-step approach using photo-induced electron/energy transfer (PET)–RAFT was first designed in [152] with zinc meso-tetra(N-methyl-4-pyridyl) porphyrin tetrachloride (ZnTMPyP) as a photocatalyst. The disparate reactivities between hydrophilic oligo(ethylene glycol) methyl ether methacrylate (OEGMA) and hydrophobic DAAm are responsible for the self-assembly of the gradient copolymers (Scheme 8) directly during the synthesis in aqueous media to form spheres and worms (Figure 4). OEGMA was preferentially incorporated into the polymer at the early stages of the polymerization, providing the colloidal stability of the further-formed amphiphilic gradient copolymer in an aqueous solution. The formation of worms occurred under a broad range of reaction conditions (at a molar content of OEGMA_500_ in a block copolymer of 3.5 to 12.5% and an overall degree of polymerization of up to 425).

**Figure 5:**
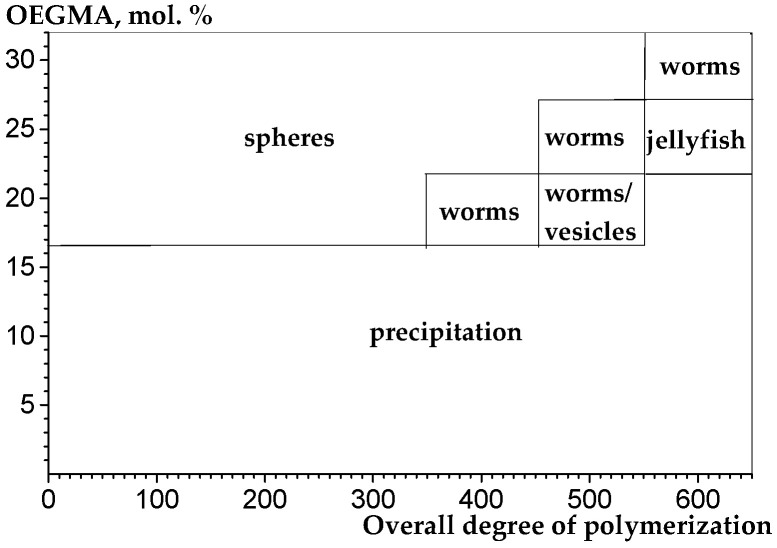
Phase diagram for a series of nanoparticles with the desired degree of polymerization and OEGMA content (mol. %) synthesized using PET-RAFT by gradient copolymerization of OEGMA and HPMA [153].

A gradual injection of core-forming monomer was used to reach the desired gradient structure of the copolymer. In this case, monomers with similar reactivities, i.e., unable to form spontaneous gradient copolymers, can be used. The aqueous gradient copolymerization-induced self-assembly of two monomer pairs, namely DMA and DAAm and OEGMA and 2-hydroxypropyl methacrylate (HPMA), was performed [153]. Altering the total copolymer degree of polymerization and solvophilic monomer composition, the spherical, worm-like micelles and vesicles were synthesized by a single-step approach (Figure 5).

**Scheme 9:**
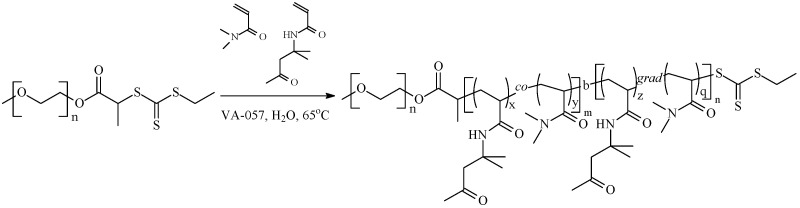
Aqueous RAFT PISA copolymerization of DMA and DAAm [154].

**Scheme 10:**
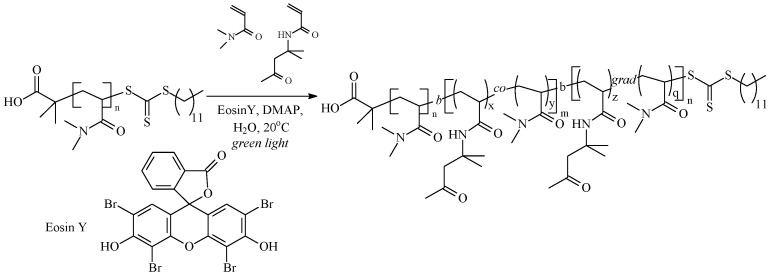
Aqueous PET-RAFT copolymerization of DMA and DAAm [154].

The choice of an appropriate macro-RAFT agent, monomer feed, and reaction media allowed Scheutz et al. to tune monomer reactivities and create gradient composition [154]. Polymerization may be initiated by conventional thermal decomposition of the radical initiator or by photoinduced electron/energy transfer using eosin Y and 4-dimethylaminopyridine (DMAP). In non-selective solvents (DMF, DMF:H_2_O = 1:1 (*v*/*v*), toluene), no self-assembly occurs in the RAFT copolymerization of DAAm and DMA in the presence of 2-(butylthiocarbonothioylthio)propionic acid, and DMA is consumed two times faster than DAAm. However, in aqueous solutions in the presence of a hydrophilic macro-RAFT agent, the reactivity of both monomers is equal, resulting in the formation of block-random copolymers at conversions before 15–20%. Later self-assembly occurs, leading to colloidal dispersion of core–shell particles with the P(DAAm-co-DMA) core-forming block, accompanied by a noticeable rise in the monomer’s reactivities. Moreover, DAAm becomes more active than DAA due to its increasingly hydrophobic locus of polymerization. Similar results are observed in the chain extension of PEO-based (Scheme 9) and PDMA-based (Scheme 10) macro-RAFT agents with DAAm and DMA. By increasing the feed ratio of DAAm, the difference in monomer reactivities deepens toward DAAm. This change affords the spontaneous generation of gradient sequences, which is inaccessible in homogeneous batch conditions.

A similar result of self-assembling during spontaneous gradient copolymerization was observed during the copolymerization of vinyl acetate (a “less activated” monomer) and acrylic acid (a “more activated” monomer) in the mixed 1,4-dioxane/water solvent under the action of poly(ethylene glycol) methyl ether (4-cyano-4-pentanoate dodecyl trithiocarbonate) at high monomer conversions [155]. The monomer conversion and monomer feed were found to affect the ability of the copolymer to self-assemble. The narrowly dispersed aggregates with an average hydrodynamic radius of about 250 nm were observed at an acrylic content of 57 mol. % in monomer feed and 77% in monomer conversion.

Gradient-induced self-assembly may occur even in bulk [156], i.e., in the absence of additional solvent, as was demonstrated for the aminoxyl-mediated spontaneous gradient copolymerization of styrene (*r*_S_ = 14.6) and *N*-vinylpyrrolidone (*r*_NVP_ = 0.04 [157]) under the action of TEMPO. The microphase-like separated structures directly form in the reaction medium, while the morphology of the resulting product is highly dependent on the monomer feed composition.

## 4. Mechanisms of Chain Activation

The realization of PISA requires either a living or reversible deactivation mechanism of the polymerization that causes the formation of amphipathic block copolymers. The majority of publications are devoted to RDRP techniques [158]. Among them, RAFT polymerization takes a dominant lead. Below, we briefly discuss the main features of RDRP and living polymerization in application to PISA.

### 4.1. Reversible Addition–Fragmentation Chain Transfer (RAFT)

**Scheme 11:**
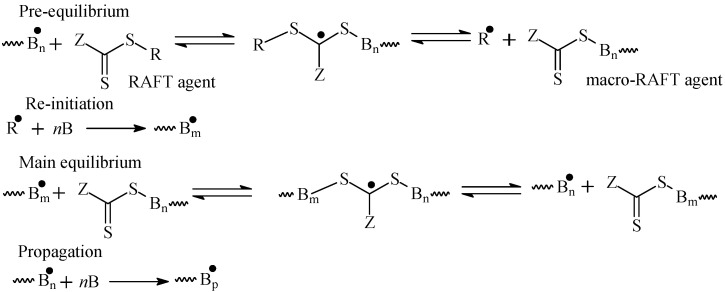
RAFT polymerization mechanism [159].

RAFT polymerization is based on the reversible chain transfer reactions proceeding via the addition–fragmentation mechanism (Scheme 11) [159,160,161,162,163,164]. The propagating radical ~B_n_^●^ arising from initiating polymerization through thermal decomposition of the initiator, its redox reaction, or PET, is involved in the reaction with RAFT agent ZC(=S)SR to form macro-RAFT agent ZC(=S)SB_n_~. During the main equilibrium, propagating radical ~B_m_^●^ participates in the reaction with the macro-RAFT agent and changes to the new macro-RAFT agent ZC(=S)SB_m_~, while releasing radical ~B_n_^●^ is involved in a propagation reaction.

Among other RDRP techniques, RAFT polymerization is the most versatile and attractive due to its low sensitivity to the functional groups in monomers and solvents and mild conditions of realization [165,166,167,168]. This allows the application of RAFT polymerization in all known PISA protocols. RAFT PISA can be performed in aqueous or organic media, including polar and non-polar solvents such as n-alkanes and mineral oils, ionic liquids, and supercritical CO_2_ [84,85,169,170,171,172,173,174,175,176]. The RAFT technique is now used for both dispersion and emulsion PISA formulations [177,178,179,180].

**Scheme 12:**
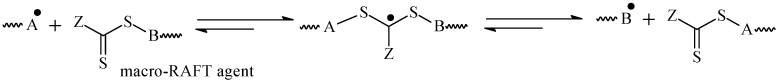
The first stage in block copolymerization of monomer A in the presence of macro-RAFT agent ZC(=S)SB~.

The following conditions should be met to implement the RAFT PISA: Firstly, according to the RAFT mechanism (Scheme 12), in the stage when the growing radical of monomer A reacts with the solvophilic macro-RAFT agent ZC(=S)SB~, fragmentation of the intermediate radical should occur via the splitting of a radical ~B^●^. As a consequence, polyacrylate or polystyrene-based macro-RAFT agents are not effective for polymerization of methacrylic monomers as the polyacrylate or polystyrene radicals are poor, leaving groups in the intermediate formed ZC^●^(SA~)SB~ compared to polymethacrylate radicals. On the contrary, due to the same reason, the polymethacrylate macro-RAFT agents are suitable for the polymerization of acrylate, styrene, and other monomers.

Secondly, the molar ratio of macro-RAFT agent and initiator is crucial for the control of MWD. Normally, it should exceed 1 to produce a polymer wi/th a narrow MWD [181,182,183]. The choice of macro-RAFT agent concentration is determined by the targeted values of *M_n_* in the growing block copolymer, while the initiator concentration should be enough to provide a suitable rate of polymerization. Hence, the researcher keeps balance between targeted *M_n_* (in the ideal case, $M_{n}=M_{RAFT}+conv.\frac{{\left[M\right]}_{0}}{{\left[RAFT\right]}_{0}}$, where *M*_RAFT_—molar mass of macro-RAFT agent, conv.—monomer conversion, [M]_0_ and [RAFT]_0_—molar concentrations of the monomer and macro-RAFT agent [184,185]), an appropriate rate, and a number of living chains while choosing the molar ratio of macro-RAFT agent and initiator.

Finally, the appropriate solvent is needed for PISA; it should be a good solvent for macro-RAFT agents or a poor solvent/non-solvent for growing blocks. The length of the macro-RAFT agent and its concentration should be enough to provide the stability of the particles of the block copolymers formed [169,170,171,172,173,186,187,188,189].

RAFT formulations are based typically on monofunctional RAFT agents ZC(=S)SR that give rise to AB-diblock copolymers and include dithiobenzoates (Z = Ph), non-symmetrical trithiocarbonates (Z = SR′, R′ poor leaving group), etc. [190,191,192]. Below, we will discuss in detail the main features of various RAFT PISA formulations designed for producing particles with desired morphology that use monofunctional RAFT agents.

Bifunctional RAFT agents that provide the growth of the chain in two directions, e.g., symmetrical trithiocarbonates (Z = SR, both R groups are leaving), enable the synthesis of ABA triblock copolymers. These RAFT agents attract less attention, probably due to their limited number. Our group has performed a systematic study of dispersion, emulsion, and mini-emulsion polymerization of styrene and acrylates using bifunctional macro-RAFT agents based on polyacrylic acid and its copolymers with *n*-butyl acrylate (BA), styrene, N-isopropylacrylamide (NIPA), sodium styrene sulfonate (SSNa), 2,2,3,3,4,4,5,5-octafluoropentyl acrylate (OFPA), and 2,2,3,4,4,4-hexafluorobutyl acrylate (HFBA) and copolymers of NIPA and BA [193,194,195,196,197,198,199,200]. Dispersion polymerization usually provides relatively good control of the MWD [193,195,197,198,199]. However, this control is lost at high conversions, and the MWD of block copolymer gets broader. In these systems, independently of the monomer-to-solvent ratio and the concentrations of macro-RAFT agent and initiator, particles of spherical morphology are formed. In emulsion polymerization, the behavior of macro-RAFT agents based on symmetrical trithiocarbonates differs from that of macro-RAFT agents based on unsymmetrical trithiocarbonates or other monofunctional RAFT agents due to the locus of the trithiocarbonate group in the middle of the chain [193,194,195]. The difference is that the polymeric product formed in the former case is characterized by bimodal MWD. This result is achieved irrespective of the chemical nature of the polymeric substituent in the macro-RAFT agent, the chemical nature of the initiator, the molar ratio of the initiator to the macro-RAFT agent, the volume ratio of water to the monomer, the monomer addition regime, etc. The coexistence of two types of block copolymers with low and high MW of core-forming block may be caused by restricted access of the trithiocarbonate group in the block copolymer with a short hydrophobic block for propagating radicals that penetrate into the particle. As a result, most of the macromolecules with short hydrophobic blocks serve as stabilizers of the particles rather than macro-RAFT agents. Additionally, in this case, polymeric particles formed in the course of emulsion polymerization reveal only spherical morphologies [193,194,195].

The recent reviews summarize the achievement of RAFT PISA in the creation of dispersions with desired morphology [201,202,203,204,205,206,207]. The crucial achievement of RAFT PISA studies is “PISA phase diagrams” (see examples in Figure 4 and Figure 5) that attribute particle morphology to the degree of polymerization of core- and shell-forming blocks [14,15,118,134,135,208]. For the given block copolymer, the phase diagram may vary for different reaction media and solid content [134,158,170,209]. The core-forming block of particles prepared in water and nonpolar solvent may become partially solvated at specific temperatures [117,210,211,212,213,214,215,216]. If the core-forming block is thermally sensitive, then the decrease or increase in temperature may cause the plasticization of particles. As an example, the core plasticization of the worm’s nanoparticles can cause the formation of a viscous gel at high polymer concentrations. Simultaneously, it can lead to reversible morphological transitions to lower-order morphologies, i.e., from worms to spheres or from vesicles to worms. The novel RAFT PISA protocols allow the synthesis of particles with more complex morphology and their morphological transitions by temperature or pH variations. To prevent changes in a particle’s morphology, the core can be cross-linked by the addition of divinylic monomer [217].

The major advantages of the RAFT technique are its unpretentiousness to monomer and solvent functionalities, along with the wide range of temperatures suitable for polymerization. The disadvantages include the necessity of the use of an initiator and the formation of sulfur-containing polymers with a ZC(=S)S-group at the end or within the chain.

### 4.2. Aminoxyl-Mediated Polymerization

**Scheme 13:**
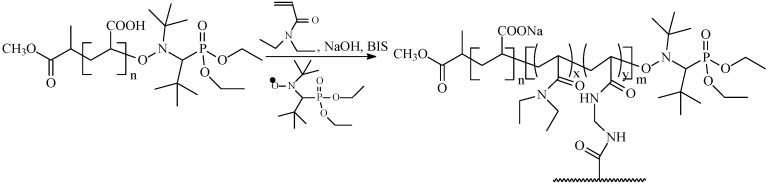
Aqueous dispersion NM-PISA of DEA and BIS [224].

**Scheme 14:**
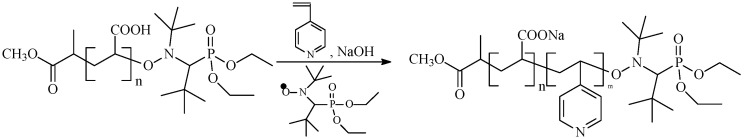
Aqueous dispersion NM-PISA of 4-vinylpyridine mediated by an alkoxyamine macroinitiator [124].

Aminoxyl-mediated polymerization (often called nitroxide-mediated polymerization, or NMP) is used more frequently than other types of stable free-radical polymerization, e.g., cobalt-mediated radical polymerization, which proceeds via different mechanisms, including reversible inhibition [218]. It is based on the reversible cleavage of terminal alkoxyamine groups. Hence, polymerization is performed at elevated temperatures. Among suitable nitroxide radicals, N-*tert*-butyl-N-(1-diethyl phosphono-2,2-dimethylpropyl) nitroxide (SG1) should be mentioned as the most appropriate for styrenic and acrylic monomers [219,220,221,222]. Charleux et al. realized the first example of NM-PISA in 2005 (Scheme 3) and have extended the NM-PISA approach to various systems [122,124,223,224,225,226,227,228]. The emulsion NM-PISA of styrene or *n*-butyl acrylate mediated by a water-soluble poly(sodium acrylate) alkoxyamine macroinitiator led to the formation of hairy nanoparticles analogous to crew-cut micelles [223]. The same hydrophilic alkoxyamine macroinitiator was used in dispersion copolymerization of N,N-diethylacrylamide (DEA) and N,N’-methylenebisacrylamide (BIS) (Scheme 13) and 4-vinylpyridine (Scheme 14) to produce temperature-responsive crosslinked nanoparticles [224] and pH-sensitive spheres, worms, or vesicles [124], respectively.

**Scheme 15:**
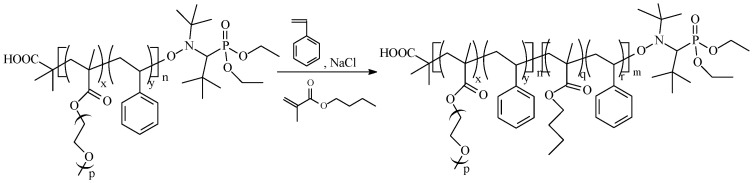
Aqueous dispersion NM-PISA of 4-vinylpyridine mediated by alkoxyamine macroin [226].

The nature of the stabilizing block was extended with a copolymer of acrylic acid and sodium styrene sulfonate terminated by alkoxyamine, which was chain extended with a random copolymer of methyl methacrylate (MMA) and styrene through the emulsion NM-PISA at 90 °C [225]. Depending on the length of the core-forming block, the successful synthesis of spheres, worms, or vesicles was performed. The brush-type macroalkoxyamine was suggested by Qiao et al. as a macroinitiator for emulsion polymerization of *n*-butyl methacrylate styrene at 85 °C (Scheme 15) [226,227,228]. Both solvophilic and solvophobic blocks contain small amounts of styrene units. Regardless of the molar mass of the hydrophobic block, spheres were formed at pH 4.2. An increase in pH led to the formation of vesicles and elongated particles, providing an unusual approach to controlling the morphology of the particles independently of the degree of polymerization of each block. When the same macroinitiator was physically adsorbed on the surface of silica particles and then employed to emulsion copolymerize the same monomers, the block copolymers self-assembled into polymer nodules randomly distributed on the silica surface [227]. The hybrid particles with dumbbell-, daisy-, or raspberry-like morphologies can be obtained by varying the macroinitiator concentration or the silica particle size. The pH value also has a significant effect on particle morphology [228]. Multipod-like particles were formed at pH values below 6, while at higher pH values, more complex unconventional morphologies were obtained, including branched worms or snowman-like vesicles.

**Scheme 16:**
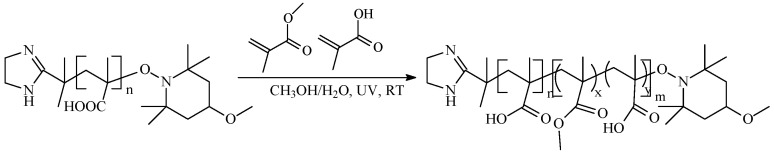
Synthesis of random block copolymer PMAA-*b*-P(MMA-*co*-MAA) through dispersion NM-PISA [229].

The research series performed by Yoshida is devoted to NM-PISA mediated by macroalkoxyamine terminated by another nitroxide radical, 4-methoxy-2,2,6,6-tetramethylpiperidine1-oxyl (M-TEMPO) [229,230,231,232,233,234,235,236,237]. Hydrophilic poly(methacrylic acid), PMAA end-capped with M-TEMPO, was used in dispersion copolymerization of MMA and MAA (Scheme 16) or ternary mixtures of MMA, MAA, and 2-(dimethylamino)ethyl methacrylate (DMAEMA). Thermal activation of such a macroalkoxyamine requires elevated temperatures (higher than 100–120 °C). Thus, Yoshida used photoactivation by UV irradiation in the presence of (4-tertbutylphenyl)diphenylsulfonium triflate. Surprisingly, the author insists on the “living” mechanism of polymerization [233], while the disproportioning reaction is more typical for methacrylate propagating radicals with nitroxide radicals (in particular, TEMPO) instead of recombination, resulting in a violation of the “living” mechanism [238,239]. Thus, the formation of pure block copolymers is unlikely, while the formation of block copolymers contaminated with homopolymers is more probable. In addition, the mixture of products may explain the formation of micron-size objects observed by Yoshida. At earlier stages of polymerization, the random block copolymer formed giant spherical vesicles and micelles of micron size. At later stages of NM-PISA, the film was obtained by the precipitation of the worm-like vesicles, and a microvillus-like structure was formed on the surface of the film that precipitated from the dispersed solution [229,230].

**Scheme 17:**
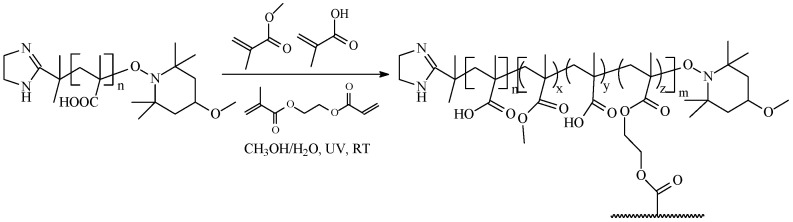
Synthesis of crosslinked random block copolymer PMAA-*b*-P(MMA-*co*-MAA-*co*-EGDMA) through dispersion NM-PISA [231].

Crosslinking of the core-forming block by ethylene glycol dimethacrylate (EGDMA, Scheme 17) caused transformation into large, contorted vesicles with a rough surface [231].

Further studies reveal the effect of the molar ratio of the MAA units in the hydrophobic block on morphology, which was transformed from spherical vesicles into fibers and then into membranes with the rise of MAA units at a constant block length [232]. The replacement of MMA with more hydrophobic isopropyl methacrylate units results in maintaining vesicular morphology. These morphology transitions were caused by the change in the critical packing shape of the random block copolymers based on the variation in the extent of the hydrophobic block chains.

Summarizing, although NM-PISA has been explored contemporaneously with RAFT PISA, it keeps out of the spotlight. NM-PISA is restricted mainly by acrylate and styrene monomers and mostly uses high temperatures for the activation of macroalkoxyamines.

### 4.3. Atom Transfer Radical Polymerization (ATRP)

**Scheme 18:**
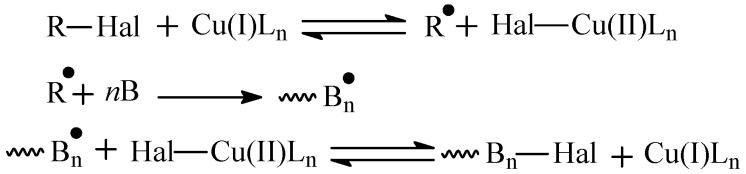
General scheme of conventional ATRP mechanism [240].

ATRP competed with RAFT polymerization over the last decades, in particular in the synthesis of the macromolecules of complex architecture [240,241,242,243,244,245,246,247,248,249,250]. It is based on the halogen exchange between dormant (~B_n_–Hal) and active (~B_n_^●^) macromolecules prompted by complexes of transition metals, typically copper (Scheme 18).

In general, ATRP is less commonly used in PISA than RAFT due to the special conditions required for ATRP implementation and the necessity to purify polymer particles from metal. These problems can be solved by using reduced copper concentrations [250]. Metal ions in different oxidation states should be present in the core of the particle to block copolymer formation that restricts ATR-PISA to dispersion polymerization.

**Scheme 19:**
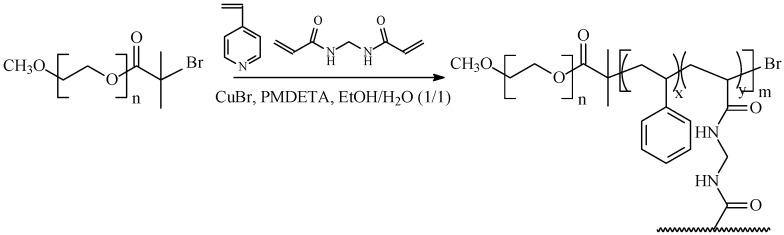
Synthesis of PEG-*b*-P4VP through dispersion ATR-PISA [246].

In the first example of ATR-PISA, dispersion polymerization of 4-vinylpyridine (4VP) in an ethanol/water mixture was performed using 2-bromoisobutyryl-terminated poly(ethylene glycol) methyl ether as a macroinitiator and a shell-forming block [246]. It resulted in the formation of diblock copolymers with relatively short blocks (overall M_n_ was about 17 kDa) and narrow MWD (Đ ~ 1.1). The stable micelles were formed when BIS was added during the polymerization stage (Scheme 19).

Later, ATRP was applied to the synthesis of triblock copolymer particles of PEO-*b*-PDMAEMA-*b*-poly(2-(methacryloyloxy)ethyl phosphorylcholine) with cross-linked methacrylate block [247] and diblock copolymer PEO-*b*-poly(2-(methacryloyloxy)ethyl phosphorylcholine) through dispersion polymerization in an isopropanol/water mixture [248]. In the latter case, the formation of diblock copolymers with a narrow MWD (Đ ~ 1.2) and an overall M_n_ of about 25 kDa was observed. An alginate-based macroinitiator was used to perform dispersion ATR-PISA of methyl methacrylate with the formation of spherical particles comprising PMMA grafted to alginate [249]. Bromo-terminated POEGMA was applied in the dispersion polymerization of benzyl methacrylate (BzMA) in ethanol and the synthesis of spherical and worm-like particles [250]. The authors succeeded in reducing copper concentration by using ICAR (initiators for continuous activator regeneration) and ATRP.

**Scheme 20:**
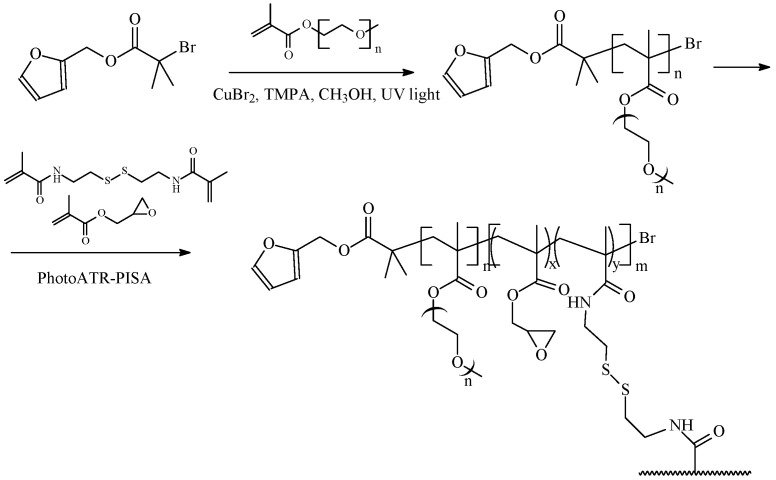
Synthesis of POEGMA-*b*-poly(glycidyl methacrylate-*co*-N,N-cystamine bis-methacrylamide) through dispersion photoATR-PISA [255].

The first comparison between initiators for continuous activator regeneration (ICAR) ATR-PISA and RAFT PISA has been performed by Zhang et al. [251]. Block copolymer particles based on poly(2-hydroxypropyl methacrylate)-*b*-PBzMA were prepared through dispersion ICAR ATRP and RAFT PISA. In both cases, the morphologies of the particles are similar and change from spheres to worm-like aggregates with block extension. However, the size of the nano-assemblies by ICAR ATRP resulted in aggregates of 4–5 times larger size than RAFT polymerization did. Another comparison between two polymerization mechanisms was carried out in the example of PEG-*b*-PS particles prepared through dispersion polymerization in ethanol [252]. However, ICAR ATR-PISA has not provided control over MWD and morphologies. The effective control for this system was achieved later [253] by chain extension of a PEG macroinitiator with styrene in the presence of PEG as a solvent. Dispersion polymerization in supercritical CO_2_ through the ATRP mechanism was described recently by Zetterlund and co-workers [254]. A bromo-terminated poly(dimethylsiloxane) was used as a macroinitiator of BzMA polymerization to produce spheres, worms, and possibly vesicles. The most recent ATR-PISA contribution is aligned with photoATRP (Scheme 20) [255].

In conclusion, ATR-PISA may find potential applications for the preparation of particles with well-defined morphology. However, RAFT PISA provides better control over MWD and particle morphology more frequently [251,252].

### 4.4. Degenerative Chain Transfer Radical Polymerization

Organotellurium-mediated radical polymerization (OMRP) has been successfully developed in the 2000s to produce polymers with narrow MWD [256,257,258,259,260]. This process is based on proceeding with both activation processes: degenerative chain transfer and thermal dissociation of labile bonds in organotellurium compounds. The first example of self-assembly using OMRP was reported by Okubo et al. [261]. PMAA-methyltellanyl (PMAA–TeMe) was used in chain extension with butyl acrylate in aqueous emulsion polymerization initiated by 4,4′-azobis(4-cyanovaleric acid) at 60 °C and alkali pH, which resulted in the synthesis of block copolymer particles with an average diameter of about 20 nm. Later, the same team reported about styrene emulsifier-free emulsion polymerization using PMAA–TeMe [262,263,264,265,266]. The control of MWD and the size of particles are affected by the polymerization temperature. The rise of the temperature from 50 to 70 °C leads to the growth of the particles from nanometer- to sub-micrometer-size and the widening of MWD from 1.5 to 4.6. Thus, homogeneous nucleation, which probably prevails at elevated temperatures, is responsible for the loss of control over uniform particles’ formation. Two-step OMRP emulsion polymerization was described in [265]. PMAA–TeMe was used first in emulsion polymerization of butyl acrylate, and then styrene was added on the second stage of polymerization, resulting in the formation of shell–corona–core particles of triblock copolymer PMAA-*b*-poly(butyl acrylate)-*b*-polystyrene with an average particle diameter of 40 nm.

Cationogenic PDMAEMA–*n*-butyl tellanyl was used in emulsion styrene polymerization [266]. The spherical particles were formed with an average diameter of 30 to 170 nm, depending on the MW of the solvophilic polymer and the rate of stirring. However, the efficiency of chain extension was not high; the polymeric tellanyl slowly consumed in the course of the polymerization, resulting in bimodal MWD of the polymer. In this case, emulsion polymerization was performed using inefficient stirring (200 rpm), in which the styrene phase floated as a layer on the aqueous phase. This approach led to the formation of polymers with better control over MWD and a smaller particle size compared to when 1000 rpm stirring was used. Kitayama et al. attribute this result to the prevalence of micellar nucleation over homogeneous nucleation at lower rates of stirring.

Unlike other RDRP techniques, OMRP has not attracted much attention. No information about dispersion polymerization or the formation of particles with morphologies other than spheres is available.

**Scheme 21:**
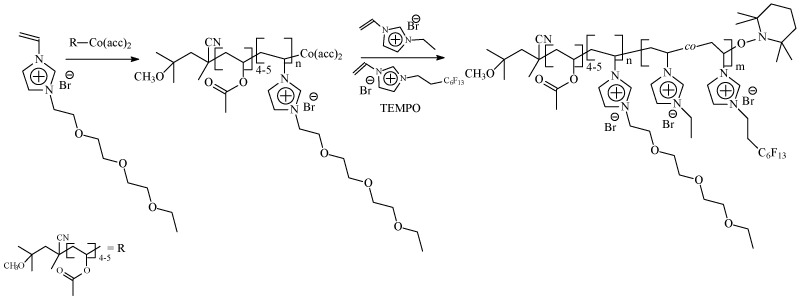
Cobalt-mediated PISA of *N*-vinyl-imidazolium-type monomers [267].

Besides OMRP, cobalt-mediated radical polymerization (CMRP) and bromine-iodine transformation (RDRP) were applied in PISA [267,268,269]. For example, *N*-vinyl-imidazolium-type monomers were involved in aqueous CMRP to produce block copolymers that self-assembled in the course of the synthesis (Scheme 21) [267]. The *N*-vinyl-imidazolium bromide with triethylene glycol pendant group was used to form a stabilizing hydrophilic block and to initiate random copolymerization of *N*-vinyl-3-ethyl- and *N*-vinyl-3-perfluorooctyl-imidazolium bromides. A subsequent anion exchange reaction substituting bis(trifluoromethylsulfonyl)-imide for bromide counter-anions leads to the formation of poly(ionic liquids), which demonstrate ionic conductivity σ_DC_ = (1 − 3)×10^−7^ S cm^−1^ and form free-standing films with mechanical properties suited for solid-phase extraction applications.

Degenerative transfer of halogen is less effective in thermally activated RDRP [270,271]. However, photoinitiation revitalized interest in this process [238]. Photo-controlled bromine-iodine transformation was applied first for the synthesis of the diblock copolymer POEGMA-*b*-PBzMA through dispersion polymerization in methanol in the presence of ethyl α-bromophenylacetate, NaI, and triethylamine. Xu et al. could observe spheres, worms, and vesicles depending on the length and the ratio of the blocks [268]. However, the degree of polymerization of the solvophilic block has not exceeded 13 units, while it was lower than 40 for the solvophobic block. When PEG block appears in the main chain (polyethylene glycol monomethyl ether 2-bromo-2-phenylacetate was used as the chain transfer agent), block copolymers with solvophobic poly(benzyl methacrylate) or poly(hydroxypropyl methacrylate) could form only spherical particles with an average diameter of 30–100 nm, depending on polymerization conditions. This approach may gain interest if block copolymers with higher MW could be obtained.

### 4.5. Various Types of Ring-Opening Polymerization

**Scheme 22:**
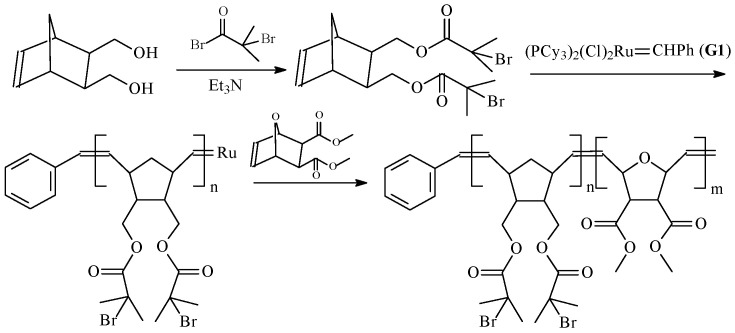
ROM-PISA of BNBE and ONBDM in toluene in the presence of Grubbs catalyst G1 [286].

Most ring-opening polymerizations (ROP), such as ring-opening metathesis polymerization (ROMP) and ROP of lactones, lactams, cyclic ethers, etc., proceed through an ionic mechanism. ROMP polymerization of cyclic monomers containing C=C bonds is initiated typically by metal alkylidene complexes M=CR(R′) such as Ru-based Grubbs catalysts of 1–3 generation (G1–G3) [272,273,274,275,276,277,278,279,280,281,282,283]. Various functional norbornenes are the most frequently used monomers in ROMP, which usually proceeds through living mechanisms, giving rise to block copolymer synthesis [284,285]. The first example of ROM-PISA was reported by Xie et al. [286]. The one-pot ROM-PISA of 2,3-bis(2-bromoisobutyryloxymethyl)-5-norbornene (BNBE) and 7-oxanorborn-5-ene-exo-exo-2,3-dicarboxylic acid dimethyl ester (ONBDM) in toluene (Scheme 22) resulted in the formation of spherical particles with R_h_ in the range of 100–200 nm.

**Scheme 23:**
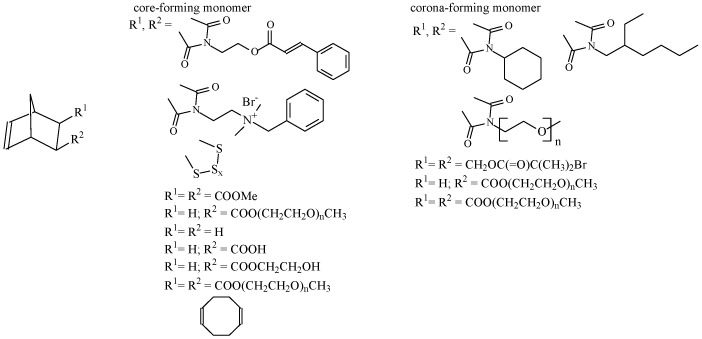
Typical core- and corona-forming monomers used in ROM-PISA [287].

Later, modified Grubbs’ catalysts were used to provide ROMP in aqueous media [287,288,289,290]. The numerous core- and corona-forming monomers with hydrophilic and hydrophobic substituents are used in ROM-PISA in organic and aqueous solutions to produce spherical block copolymer particles [287,288,289,290]; some of them are given on Scheme 23.

**Scheme 24:**
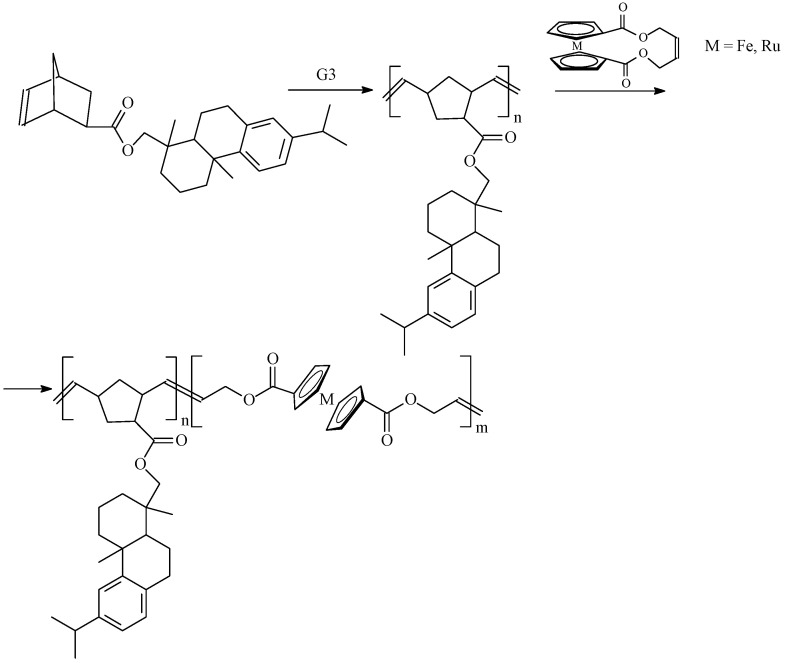
Scheme of polymerization-induced crystallization-driven self-assembly through sequential ROMP of functional norbornene and ruthenocenyl olefin monomers [290].

Polymer particles of AB- and ABC-block copolymers with a crosslinked core obtained through ROM-PISA of 2,3-bis(2-bromoisobutyryloxymethyl)-5-norbornene, exo-N-(cinnamoyloxyethyl)-7-oxanorborn-5-ene-2,3-dicarboximide, and 7-oxanorborn-5-ene-exo,exo-2,3-dicarboxylic acid dimethyl ester are described in [291]. The ROM-PISA of a dicyclopentadiene with a non-norbornene-based macromolecular chain transfer agent prepared by ROP of ε-caprolactone followed by reaction with cis-2-butene-1,4-diol to prepare crosslinked polymer nanoparticles is described in [292]. Higher-order morphologies are reported for block copolymer poly(5-methoxycyclooctene)-*b*-polyferrocene [288]. Enzyme-responsive block copolymer nanoparticles with the peptide sequence GPLGLAGGWGERDGS attached to norbornene dicarboximide units are described in [293,294]. The combination of PISA and crystallization-driven self-assembly through one-pot sequential ROMP to prepare nano-objects based on a crystalline poly(ruthenocene) motif is realized in [290] (Scheme 24). Fiber-like and platelet micelles were observed, depending on polymerization conditions.

Recently, a morphological evolution mechanism based on an in situ vesicle–vesicle fusion process that forms tubular polymersomes was studied for block copolymers comprising tertiary amine functional polynorbornene or a PEGylated polynorbornene as a stabilizing block and poly(norbornene imide ethylene glycol monomethyl ether) as a core-forming block [295]. Block copolymer was synthesized through aqueous dispersion (ROM-PISA), and the transformation of vesicles into tubular polymersomes was observed throughout polymerization.

**Scheme 25:**
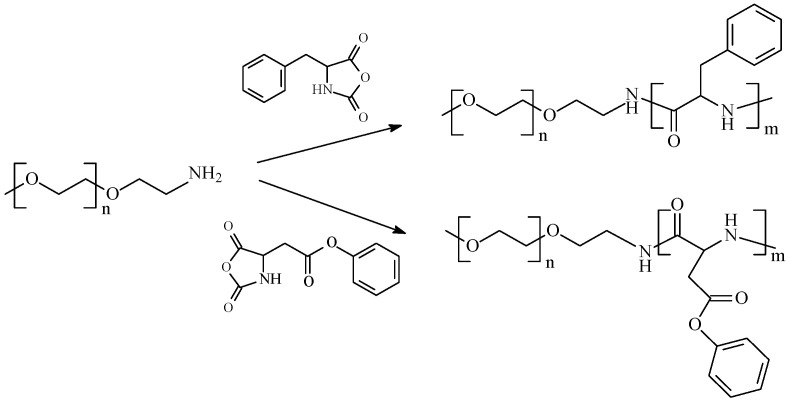
NCAs ring-opening PISAs for the formation of polypeptides [297].

The controlled synthesis of polypeptides can be performed by ROP of α-amino acid N-carboxyanhydrides (NCAs) [296]. A new PISA methodology called NCA-PISA to prepare polypeptide spherical or vesicular particles with a diameter of 0.5–1.2 μm has been developed recently [297]. According to that strategy, α-amino acid NCAs of L-phenylalanine monomer or NCAs of L-aspartic acid β-benzyl ester are polymerized by methoxypolyethylene glycol amine (Scheme 25). This polymerization is realized under much milder conditions without the involvement of high temperatures, an oxygen-free environment, catalysts, or macromolecular chain transfer agents. Vesicular morphology can be finely regulated by the hydrophilic/hydrophobic ratio and solid content. The heterochain macromolecules formed in this case can be degraded by treatment with trypsin.

There are numerous examples of NCA-PISA, including aqueous polymerization [158,201,298,299,300,301]. This strategy is applicable to various macroinitiators and NCA monomers. Along with common morphologies such as spheres and worms, needle-like structures [299] or long rods [300] were observed. Grazon et al. suppose that the secondary structure of the polypeptides is the main driving force to stabilize the anisotropic rod-like nanostructures.

**Scheme 26:**
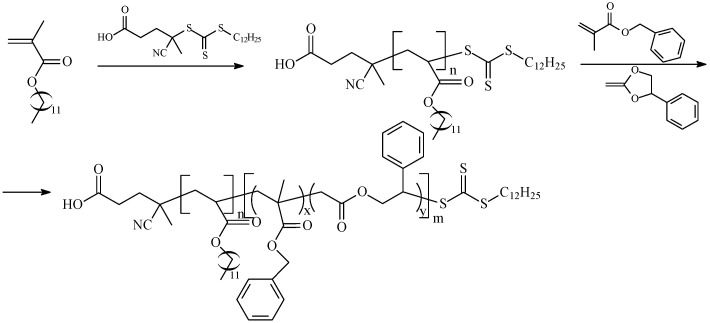
Radical ring-opening PISA process to produce poly(lauryl methacrylate)-*b*-poly(benzyl methacrylate-*co*-2-methylene-4-phenyl-1,3-dioxolane) diblock copolymer nanoparticles [304].

The radical mechanism of ROP can be realized in the case of cyclic ketene acetals [302,303]. For example, dispersion RAFT copolymerization of benzyl methacrylate with 2-methylene-4-phenyl-1,3-dioxolane or 5,6-benzo-2-methylene-1,3-dioxepane was in heptane at 90 °C (Scheme 26). Later, the same cyclic ketene acetals were involved in aqueous emulsion polymerization using lauryl methacrylate as a comonomer and poly(oligo(ethylene glycol) methyl ether methacrylate-based macro-RAFT agent as a solvophilic block [304]. The introduction of ester groups in the backbone confers polymer degradation properties.

### 4.6. Living Anionic Polymerization

Shortly after the discovery of PRIMSA in the early 2000s, living anionic polymerization-based PISA (LA-PISA) was scarcely successful due to restrictions on experimental conditions and monomers used. The recent interest in LA-PISA may be caused by the overwhelming success of living anionic polymerization in the synthesis of precise macromolecular architectures [305,306,307,308,309]. However, the defined order of monomer addition and the limited combinations of monomers with similar activities able to form block copolymers without changing the activity of growing macroanion have limited the monomers to styrenes and 1,3-dienes [310,311,312]. The formation of block copolymers of polyisoprene (shell-forming block) and polystyrene (core-forming block) or its derivatives via dispersion LA-PISA in heptane initiated by *n*-butyl lithium is described in [310]. The addition of divinyl benzene at the final stage of polymerization results in crosslinking of the core-forming block and freezing of the morphology of the particles. Variation of MWs of both blocks, the ratio of their MWs, weight solid content, and nature of substitutes in styrene monomer provide efficient control of morphologies. Wang et al. have observed spheres, worms, and vesicules. All-styrenic diblock copolymer synthesized through LA-PISA is described in [311]. Solvophilic poly(*p*-*tert*-butylstyrene) was synthesized first in heptane, followed by dispersion polymerization of styrene. A similar transformation of morphologies was observed due to the change of MW and the ratio of MWs of the blocks. Zhou et al. have plotted the phase diagram defining the following regions of formation: spheres, worms, vesicles, their mixtures, or irregular morphology. Living anionic polymerization-induced cooperative assembly (LA-PICA (PICA means a synthesis of a mixture of block copolymer and homopolymer using the mixture of a living macro-initiator and a small-molecule initiator taken in different ratios. PICA is discussed in detail in the Section 6.2)); to prepare higher-order morphologies (porous vesicles, sponges, cubosomes, and hexosomes), it is suggested in [312]. Polymerization of isoprene was initiated by *n*-butyl lithium in heptane. On the second stage, dispersion polymerization of styrene was performed in a TMEDA/heptane solution in the presence of a lithium macro-initiator and a small-molecule initiator. The simultaneous formation of homopolymers and block copolymers provokes the formation of complex morphologies.

Summarizing, numerous possibilities of chain activation in PISA processes allow producing a wide variety of morphologies from monomers of various chemistry. Among RDRP techniques, RAFT polymerization is most preferable due to the widest range of available monomers, reaction conditions, and accessible morphologies. Living anionic, ROM, and ROMP PISA protocols can be applied to a restricted number of monomers; however, these monomers are commonly unable to polymerize through other mechanisms.

## 5. Mechanisms of Particle Formation

The PISA process involves the formation of amphiphilic block copolymers that undergo self-assembly during the polymerization process. The mechanism of particle formation depends on the initial state of the reaction mixture—whether it is homogeneous or heterogeneous.

In the first case (dispersion polymerization), a solvophilic macroinitiator launches the polymerization of a solvophobic monomer that is soluble in the reaction mixture. Therefore, solution polymerization of a solvophobic monomer takes place first, but at a critical degree of polymerization of the growing block, it becomes insoluble, and in situ micellar nucleation occurs. The medium becomes turbid at a specific time, called the onset of nucleation [204]. Nucleation is followed by the diffusion of the monomer into the micelles, and the unreacted monomer can act as a co-solvent for the insoluble block within the swollen micelles. The mechanism of further growth activation of the block copolymer growth depends on the type of polymerization [10,13,14,15,170]. For RAFT polymerization, additional initiation is needed to produce active species. This initiation can take place either inside or outside of the particles. If it occurs outside, the oligomeric radicals need to get inside the particle in order to continue the polymerization process. Typically, the solution polymerization proceeds more slowly than after micellar nucleation, due to the relatively high local concentration of monomer within the swollen nanoparticles [203,206]. At this stage, the dispersion becomes progressively more heterogeneous and opaque, and further PISA proceeds while the number-average diameter of the particles either remains constant or slightly grows [16,118,170,207].

In contrast to dispersion polymerization, emulsion polymerization is an initially heterogeneous polymerization process. In a simple case, an ab initio system consists of water, an initiator, a water-insoluble monomer, and a surfactant. Emulsification leads to the formation of the monomer droplets, while the surfactant can form micelles itself or stabilize the monomer droplets. Particle formation can occur by three mechanisms: micellar entry, homogeneous nucleation, and droplet nucleation [313]. The first examples of RDRP in emulsion, e.g., the addition of a small-molecule RAFT agent or alkoxyamine to emulsion polymerization, typically led to the loss of colloidal stability and control of MW and MWD due to the presence of the water-insoluble RAFT agent in monomer droplets [128,314,315,316,317]. The use of hydrophilic or amphiphilic macroinitiators (macroalkoxyamine) or macro-RAFT agents helped to overcome these problems.

If no conventional radical initiator is needed, then the mechanism of particle formation in emulsion PISA is similar to that discussed above for dispersion polymerization [128,318]. However, in RAFT, a polymerization initiator is used to generate radicals. Thus, both ways of nucleation—micellar and homogeneous—become possible depending on the efficiency of the macro-RAFT agent, its concentrations, and the concentration of the initiator [13,319].

The first studies of particle formation mechanisms on the example of PAA-*b*-PS synthesis caused rather interesting discussion. In these studies, non-symmetrical trithiocarbonates were used to prepare short hydrophilic PAA (macro-RAFT agent) or amphiphilic block copolymers with short hydrophilic PAA blocks (5–10 units) and short hydrophobic PS blocks (5–10 units), followed by polymerization of styrene [121,317,320,321]. In some experiments, the number of RAFT molecules per particle exceeds the value of the typical aggregation number [317,320]. In other experiments, these values were similar [321]. The key factor in these experiments is the concentration of the macro-RAFT agent.

Ferguson et al. proposed that at the early stages of the polymerization block, copolymers formed with a hydrophilic block of PAA and a short hydrophobic block of PS [320]. It is essential that, to avoid the presence of hydrophobic monomer droplets during the particle formation step, a very slow, starved feed of the styrene monomer be used. These block copolymers undergo self-assembly to form micelles. It is critical that the active thiocarbonylthio-moiety (ZC(=S)S–) is located inside the micelle to facilitate the polymerization of hydrophobic monomers. It is supposed that the hydrophobic block in micelles grows due to monomer diffusion, and the size of the so-formed particle increases. To stabilize the increasing surface of the particle, molecules of block copolymers that are not hydrophobic will migrate from other un-entered micelles and adsorb on the surface of the particles. This idea correlates well with the effect of macro-RAFT agent concentration on particle size and the number of particles. However, when the hydrophobic block of a block copolymer becomes long enough, the migration of macromolecules between micelles stops. After all the labile blocks are adsorbed on the surface of particles, the nucleation stage is finished. The number of particles slightly changes or even remains constant throughout monomer conversion. This mechanism is similar to the micellar entry mechanism in conventional emulsion polymerization, except the surface-active block copolymer increases its hydrophobicity due to the growth of hydrophobic block. The newly formed particles then act as a seed for the new feed of hydrophobic monomers added to the reaction at a higher feed rate.

In the experiments of Ganeva et al., no evidence could be found for fast exchange (migration) of block copolymers between micelles [321]. The ability of the block copolymers to migrate depends on their overall hydrophobicity, resulting in equilibration times in the range of seconds to hours. The surface-active properties of PAA and PAA-*b*-PS macro-RAFT agents allow us to determine the CMC and compare the polymerization kinetics below and above the CMC. A detailed study of emulsion styrene polymerization in the presence of PAA-*b*-PS macro-RAFT agent above CMC led to the conclusion that the exchange of block copolymers between micelles is slow relative to particle nucleation, i.e., each micelle can become a particle. Variation of styrene feed (slow feed, followed by fast styrene feed; shot of small feed, followed by fast styrene feed; fast styrene feed throughput polymerization) has no visible effect on the particle size and their number opposite to batch reaction when the entire amount of styrene is added to the reaction mixture prior to polymerization. The nucleation of all micelles is possible if a sufficient number of initiator radicals is generated over the micelle lifetime. For systems with the most hydrophobic (least labile) block copolymer, a smaller amount of initiator is needed to nucleate all micelles than for the less hydrophobic (more labile) block copolymer. If a low initiator concentration is used, then only some of the micelles can be nucleated. Other un-entered micelles can serve as reservoirs and migrate to stabilize newly formed particles.

When the macro-RAFT agent concentration is below CMC, Ganeva et al. [321] observe similar results as described in [320]. In this case, the homogeneous-coagulative nucleation mechanism is most probable. Here, the initiator radicals react with monomers in the aqueous phase and grow beyond a critical degree of polymerization; after that, these chains form precursor particles, which then swell with monomers and are rapidly stabilized by macro-RAFT agents, transforming into final particles.

Summarizing, the correct choice of experimental conditions can increase the prevalence of micellar nucleation over homogeneous nucleation and the formation of block copolymer particles with a narrow MWD and size distribution.

## 6. Novel Types of PISA

The recent progress in RDRP techniques, in particular the development of new initiating systems and the synthesis of new complex macromolecular architectures, has caused the creation of new PISA protocols and tools that provoke self-assembly during polymerization. At present, the following PISA processes have been described [202,312,322,323,324,325,326,327]:

– polymerization-induced thermal self-assembly, PITSA;
– polymerization-induced cooperative assembly, PICA;
– polymerization-induced electrostatic self-assembly, PIESA;
– polymerization-induced hierarchical self-assembly, PIHSA;
– polymerization-induced particle/surface self-assembly, PIPA/PISSA.

**Figure 6:**
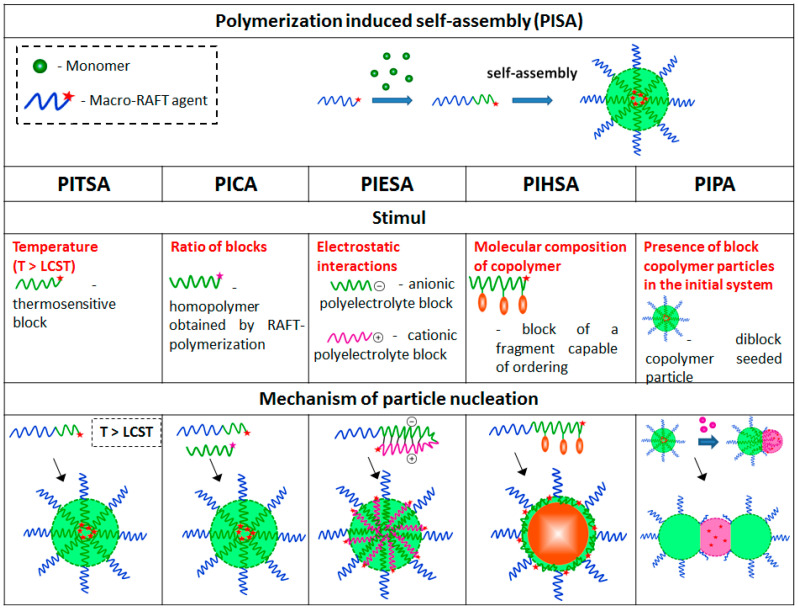
The stimuli provoking PISA and the mechanism of particle nucleation.

The main features of these PISA processes are summarized in Figure 6. They differ by the stimulus that causes the self-assembly of growing species and the mechanism of particle nucleation. Their common features are the formation of stable dispersions in the absence of an external stabilizer and the living mechanism of polymerization. Below, we will discuss the new PISA processes in detail.

### 6.1. Polymerization-Induced Thermal Self-Assembly (PITSA)

Polymerization temperature may affect the morphology of the particles formed if one of the blocks is thermosensitive. In this case, the decrease in temperature after polymerization is complete may cause a transition between various morphologies if the reaction temperature is above the critical solution temperature (LCST or UCST) [188,328,329,330,331,332]. The temperature has an impact on the kinetic features of the polymerization and the particle nucleation mechanism [134,333,334]. To participate in PITSA, a block or gradient copolymer should contain segments of thermosensitive polymer that allow designating these copolymers as smart objects. Typically, polymerization is performed in aqueous media above LCST under the action of hydrophilic polymers (macroinitiators or macro-RAFT agents). The formation of block copolymers is accompanied by the loss of solubility of the growing thermosensitive block, resulting in the formation of polymer particles stabilized by hydrophilic block. The core of the polymeric particles becomes the locus of the further polymerization reaction.

**Scheme 27:**
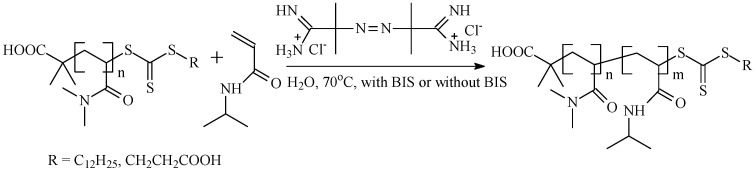
Chain extension of PDMA-based macro-RAFT agent with NIPA above LCST [119].

**Figure 7:**
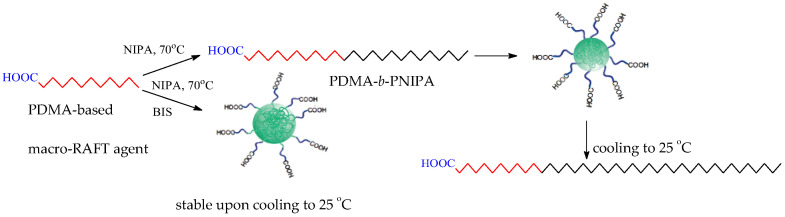
Example of RAFT PITSA in the presence and absence of cross-linker BIS [119].

In the first example of PITSA, hydrophilic PDMA with a trithiocarbonate group was chain extended with NIPA in an aqueous solution above LCST (Scheme 27) [119]. Self-assembly of block copolymers resulted in the formation of spherical particles above LCST (Figure 7). However, the decrease in temperature below LCST has transformed the dispersion into a solution, in contrast to classical PISA, which is characterized by the formation of stable dispersions independently from the temperature. To protect the particles formed during PITSA from dissolution, a cross-linker, BIS, was added at the stage of NIPA polymerization, resulting in the formation of a cross-linked core and hence stable dispersions incapable of dissolving upon cooling.

**Scheme 28:**
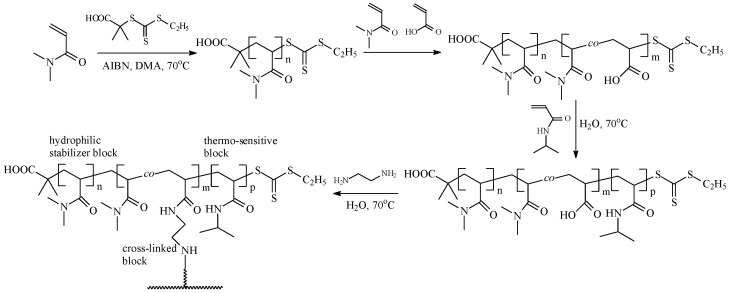
Synthesis of PNIPA-based thermosensitive block copolymer self-assembled particles above LCST [322].

A similar approach to performing RAFT PISA on NIPA was used in [322]. Block-random copolymer of PDMA-*b*-P(DMA-*co*-AA) terminated with SC(=S)SC_2_H_5_ served as a hydrophilic macro-RAFT agent (Scheme 28). However, in the final stage, cross-linking was performed using ethylene diamine, which reacts with the carboxylic groups of acrylic acid units. As a result, the block polymer particle stable above and below LCST was obtained; it contained PDMA linear blocks in a shell, a block of random copolymer of DMA and AA cross-linked through ethylene diamine as a corona, and a PNIPA block as a core. The morphology of non-cross-linked block copolymer particles transformed with the growth of the degree of polymerization of PNIPA from micelles to linear and branched warms and finally to vesicles.

**Scheme 29:**
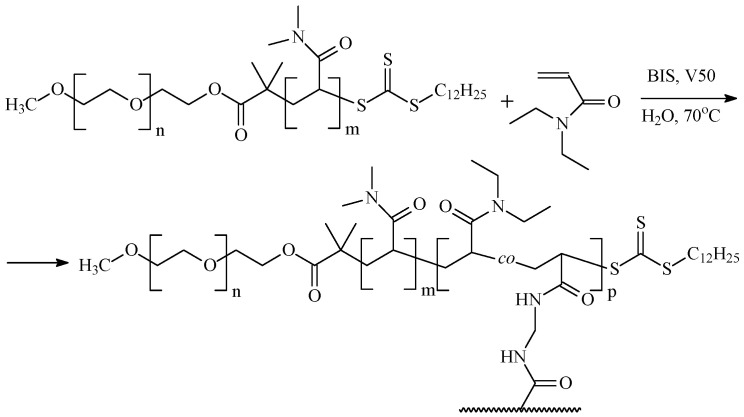
Synthesis of PEG-*b*-PDMA-*b*-P(DEA-*co*-BIS) nanogels [335].

**Scheme 30:**
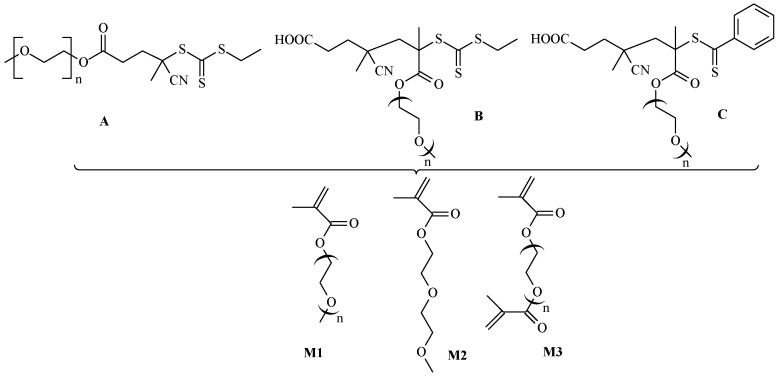
Synthesis of nanogels based on PEG-terminated trithiocarbonate (**A**), PEGMA-terminated trithiocarbonate (**B**), POEGMA-terminated dithiobenzoate (**C**), oligo(ethylene glycol)methacrylates (M1 and M2), and oligo(ethylene glycol) dimethacrylate (M3) [336].

**Figure 8:**
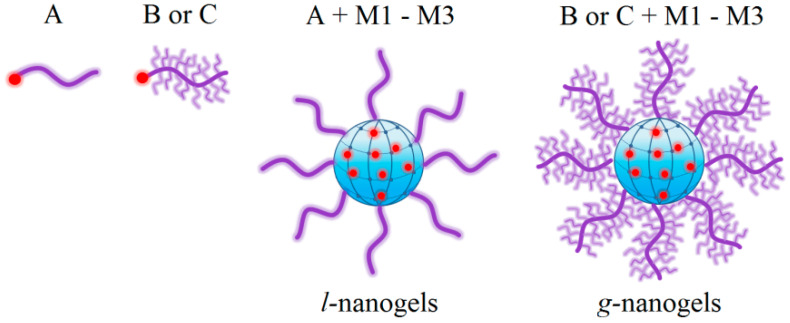
Schematic image of linear (*l*) and graft (*g*) nanogels formed in the course of RAFT PITSA of monomers M1–M3 under the action of macro-RAFT agents A–C. The structure of the monomers and macro-RAFT agents is given in Scheme 30 [336].

**Scheme 31:**
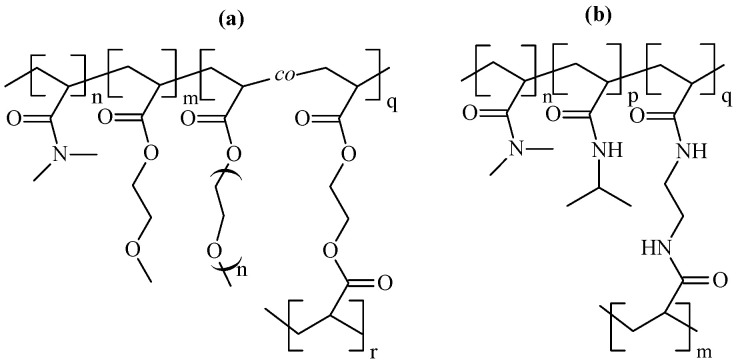
Synthesis of PDMA-*b*-P(2-methoxyethyl acrylate-*co*-OEGA-*co*-DEGA) (**a**) and PDMA-*b*-P(NIPA-*co*-BIS) (**b**) nanogels [337].

**Scheme 32:**
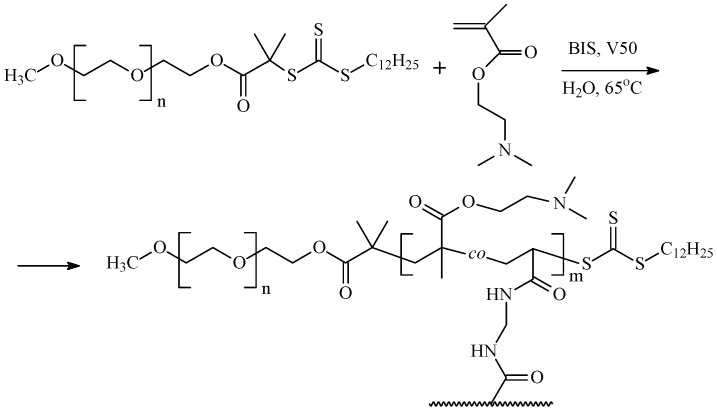
Synthesis of PEG-*b*-P(DMAEMA-*co*-BIS) nanogels [338].

PITSA is a suitable method to synthesize thermosensitive nanogels. To anchor the particle structure, the core-forming block should be cross-linked during the polymerization step. Typically, bifunctional monomers (BIS; di(ethylene glycol) methyl ether methacrylate (DEGMA) or di(ethylene glycol) methyl ether acrylate (DEGA) as examples) are copolymerized with the monomer that forms the thermosensitive block. Thus, the formed nanogels are unable to disperse into individual macromolecules upon cooling the dispersions below LCST. Their response to the temperature manifests itself as a shrinkage of nanogel above LCST and a swelling of nanogel below LCST. Among the monomers that are responsible for the formation of thermosensitive blocks, NIPA, DEA (Scheme 29) [335], OEGMA (Scheme 30, Figure 8) [336], oligo(ethylene glycol) methyl ether acrylates (OEGA, Scheme 31) [337], and DMAEMA (Scheme 32) [338] are the most popular.

**Scheme 33:**
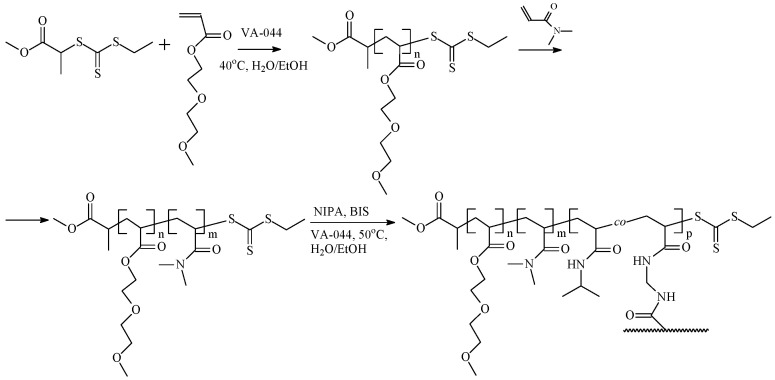
Synthesis of PDEGA-*b*-PDMA-*b*-P(NIPA-*co*-BIS) nanogels [340].

This approach is applied to water-soluble monomers. The incorporation of a number of hydrophobic monomers requires the addition of a water-miscible co-solvent such as alcohol (ethanol, isopropanol, and *tert*-butanol). Evidently, the solubility of the polymer depends on the solvent. The addition of a low amount of non-solvent (ethanol, methanol, THF, DMF, acetone, etc.) leads to a decrease in the LCST of PNIPA from ~32 °C to lower temperatures [339]. Therefore, block copolymer self-assembly can start at lower temperatures than it does in an aqueous solution. Scheme 33 illustrates the synthetic route to the formation of PDEGA-*b*-PDMA-*b*-P(NIPA-*co*-BIS) nanogels [340]. Both PDEGA and PNIPA are thermosensitive blocks. In an aqueous solution, PDEGA loses its solubility at 40 °C; however, it is soluble in a water/ethanol (60/40 wt%) mixture. Thus, the sequential RAFT solution polymerization of DEGA and DMA in water/ethanol (60/40 wt%) mixture followed by dispersion copolymerization of NIPA and BIS in water/ethanol (65/35 wt%) mixture resulted in the formation of nanogels with the corona block of PDEGA, the shell block of PDMA, and the cross-linked core of P(NIPA-*co*-BIS).

**Scheme 34:**
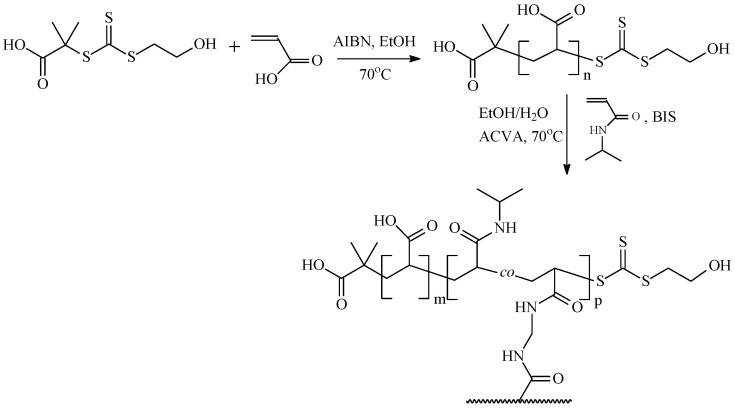
Synthesis of PAA-*b*-P(NIPA-*co*-BIS) nanogels [341].

A similar mixed solvent was used for the synthesis of nanogels through RAFT dispersion polymerization with the shell block of PAA and cross-linked core of P(NIPA-*co*-BIS) (Scheme 34) [341].

### 6.2. Polymerization-Induced Cooperative Assembly

**Scheme 35:**
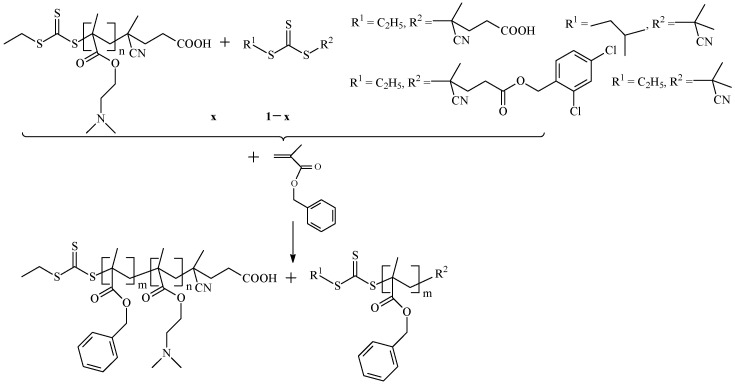
PICA of BzMA in the presence of macro-RAFT agents and small-molecule RAFT agents [342].

Polymerization-induced cooperative assembly (PICA) was first introduced in 2017 by An et al., who suggested the synthesis of a mixture of block copolymer and homopolymer using a mixture of macro-RAFT and small-molecule RAFT agents taken in different ratios (Scheme 35) [342]. In a typical example, dispersion polymerization of BzMA was conducted in ethanol using PDMAEMA as a macro-RAFT agent, which resulted in the formation of a stable dispersion of a mixture of PDMAEMA-*b*-PBzMA and PBzMA. The morphology of the polymer particles depends on the molar ratio and shifts from spheres to worms and vesicles.

The idea of PICA is based on the findings that blending amphipathic block copolymer with solvophobic homopolymer may influence the morphologies in bulk [343,344], thin films [345,346], and solution [32,347]. In the case of amorphous polymers, the homopolymer should have a lower molecular weight than the core-forming solvophobic block of the block copolymer. The study of the blending of dispersion of PS-*b*-PAA spherical particles (prepared through the addition of water to a DMF solution of block copolymer) and PS solution resulted in an increase in the size of the particles and their dispersion without change in morphology [32]. However, if the block copolymer particles have vesicular or cylindrical morphology, the addition of PS leads to a morphological transition to spheres. It was proposed that this transition was caused by the phase separation of PS homopolymer from the solvophobic PS core of block copolymer particles. An opposite observation was described in [347]. Ouarti et al. have studied the influence of PS on the morphology of block copolymer micelles (prepared by direct dissolution in heptane at elevated temperature) comprised of linear and cyclic PS-*b*-PI. It was demonstrated that the chain architecture is also a key parameter for controlling the organization of micelles formed in solution. For a polymer concentration of 3 mg/mL, the linear block copolymer self-organizes into spherical micelles, while the cyclic block copolymer forms cylindrical micelles. PS and PI chains constitute the core and the corona of these micelles, respectively, due to the different affinity of the blocks for heptane. Upon addition of PS, a morphological transition, from spheres to cylinders for the linear copolymer and from cylinders to vesicles for the cyclic copolymer, is observed. Consequently, the PS homopolymer added is “solubilized” into the micellar core. The fact that the addition of small amounts of PS (2 or 5 wt%) has such a drastic effect on the micellar morphology suggests that the block copolymer composition studied here is close to a phase transition in the phase diagram of the system. It was proposed that to accommodate the increased asymmetry between hydrophilic and hydrophobic volumes per chain, interfacial curvature increases with increasing the insoluble fraction while respecting the constraints imposed by the connectivity between the two blocks and the incompressibility of the core domain.

Thus, the simultaneous formation of dispersion of block copolymer particles via PISA and homopolymer corresponding to core-forming block may cause morphologic transitions directly in the course of the synthesis.

To achieve the simultaneous formation of a block copolymer and a homopolymer, the macroinitiator and small-molecule initiator (ATRP or aminoxyl-mediated polymerization) or macro-RAFT agent and small-molecule RAFT agent (RAFT polymerization) should be used. If the synthetic conditions are chosen properly, the amphipathic block copolymer is formed alongside the solvophobic homopolymer [342,348,349,350,351]. Opposed to block copolymer, the homopolymer formed is unable to self-stabilize after it loses its solubility. Evidently, the homopolymer should lose its solubility before the block copolymer does due to the presence of a solvophilic block in the latter, which increases the solubility of the block copolymer. Therefore, the loss of homopolymer’s solubility should induce the adsorption of block copolymer onto the solvophobic polymeric aggregates, resulting in the formation of polymer particles with the core consisting of homopolymer and solvophobic block of block copolymer and the shell of solvophilic block. The core of the particles may further swell due to the appearance of a monomer producing a homogeneous “solution”, resulting in the further growth of both the core-formed block and the homopolymer.

**Scheme 36:**
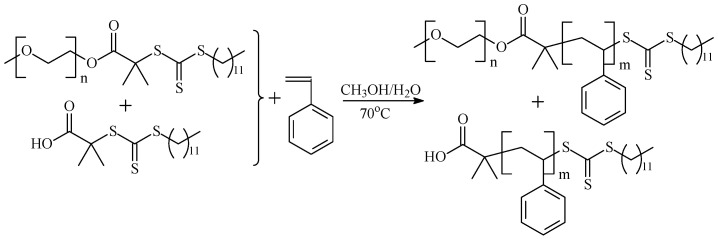
PICA of styrene in the presence of macro-RAFT agents and small-molecule RAFT agents [348].

The dispersion polymerization of styrene in the water/methanol (80/20 *w*/*w*) mixture in the presence of an equimolar mixture of 2-(dodecylthiocarbonothioylthio)-2-methylpropanoic acid (C_12_H_25_SC(=S)SC(CH_3_)_2_(COOH)) as a small-molecule RAFT agent and PEG-terminated trithiocarbonate as a macro-RAFT agent (Scheme 36) at 70 °C resulted in the formation of particles of various morphology [348]. With the increase in degree of polymerization (DP) of PS and PS block in PEG_45_-*b*-PS, the morphology changed from spheres (DP = 49) to vesicles and spheres (DP = 99), to porous vesicles and spheres (DP = 146), and finally to porous vesicles and porous nanospheres (DP = 218 and 278). All the synthesized polymers have a narrow MWD (Đ < 1.15). In the absence of PS homopolymer, the vesicles were formed with an average membrane thickness of 35 nm. The addition of PS ([PS]/[PEG-*b*-PS] < 1) caused the formation of pores in the membrane, and the thickness became non-uniform. When [PS]/[PEG-*b*-PS] = 1, the formation of porous nanospheres was also observed.

Formation of the porous nanoparticles has been observed recently in the seeded dispersion RAFT polymerization of styrene using the dispersion of PEG_45_-b-PS_151_ macro-RAFT agent in a water/methanol mixture (1/4 *v*/*v*) as a seed. Zhou et al. proposed that the formation of the porous nanospheres is due to the encapsulated styrene within the seed vesicles. This encapsulated monomer swells the seeded vesicles and therefore leads to the formation of porous nanospheres [352]. Tan et al. [348] supposed that the formation of porous vesicles or nanospheres could be attributed to a similar reason. To prove it, the synthesized vesicles PEG_45_-*b*-PS_192_ were swelled with styrene or toluene in a methanol–water mixture (40% *w*/*w* water). After this swelling procedure, the vesicles were converted into porous vesicles and porous nanospheres.

**Scheme 37:**
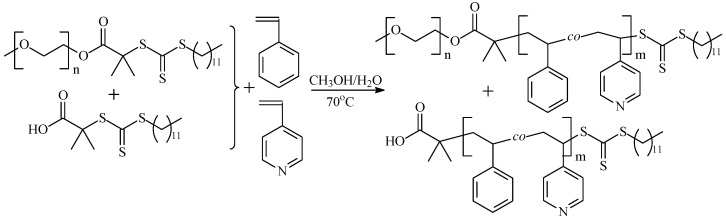
Copolymerization of styrene and 4-vinyl pyridine in the presence of macro-RAFT agents and small-molecule RAFT agents through the PICA approach [350].

The novel idea of PICA was suggested in [350]. Huang et al. have performed the copolymerization of solvophobic monomer styrene and solvophilic monomer 4-vinyl pyridine (4VP) in the water/methanol mixture in the presence of macro-RAFT and small-molecule RAFT agents (Scheme 37). By varying the molar ratios of solvophobic and solvophilic monomers and RAFT agents, polymer nano-objects of different morphologies (porous vesicles, large compound vesicles (LCV), and lamellae) were formed. In general, increasing the amount of P(St-*co*-4-VP) increases the number of domains on the vesicle surface, promoting the formation of LCVs. The proposed mechanism of LCV formation is as follows: Block and random copolymers are formed during PISA copolymerization of styrene and 4-VP, mediated by two RAFT agents. The incorporation of 4-VP increases the solvophilicity of P(St-*co*-4-VP), thus shifting P(St-*co*-4-VP) from the inner part of the vesicle membrane to the surface to form domains. As the polymerization proceeds, the size of the P(St-*co*-4-VP) domains arising from random copolymers without a stabilizing block of PEG increases. To lower the interfacial energy inside and within vesicles, vesicles collapse and aggregate to form LCVs. As polymerization further proceeds, reorganization of polymer chains occurs, and the internal structure becomes more and more compact. This proposed mechanism indicates that both the small-molecule RAFT agent and the solvophilic monomer 4-VP are crucial for preparing LCV.

**Scheme 38:**
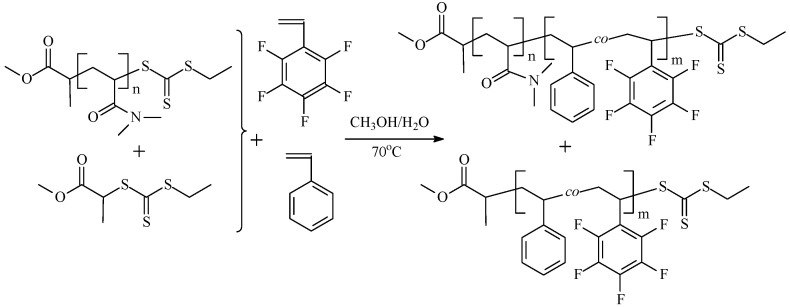
Copolymerization of styrene and 4-vinyl pyridine in the presence of macro-RAFT agents and small-molecule RAFT agents through the PICA approach [349].

A similar idea was realized by Lv et al. through RAFT dispersion alternating copolymerization of styrene and pentafluorostyrene in 2% *v*/*v* toluene/ethanol using a PDMA-based macro-RAFT agent and 2-ethylsulfanylthiocarbonylsulfanylpropionic acid methyl ester as a small-molecule RAFT agent (Scheme 38) [351]. The kinetics of PICA and PISA processes were compared, and the apparent rate constants observed in both systems were essentially the same. For the PISA process, only macro-RAFT agents were used. The PICA process has the typical features of “living” polymerization, i.e., the MWs of the polymers increased with the progress of monomer conversion, and the dispersion of polymers was low. Thus, a uniform distribution of polymer chains within the particles is expected. As the degree of polymerization of the core-forming block/solvophobic copolymer increased, morphological transitions followed the sequence of a sphere–worm–vesicle–large compound vesicle–sponge–inverse bicontinuous mesophase. Mixed inverse bicontinuous mesophases were observed in all PICA syntheses conducted at different RAFT agents’ molar ratios *x* (0.1, 0.3, and 0.6) and solid contents (10, 20, and 30% *w*/*v*), among which the mesophases obtained at *x* = 0.3 and 30% *w*/*v* showed the highest order.

Recently, the molecular dynamics simulation has suggested that the presence of the solvophobic homopolymer in the core of the particles results in the “effect of retarding chain growth”, which slows down the polymerization kinetics [347]. The simulations also confirmed the possibility of a transition from lower-order to higher-order morphologies. Simultaneously, the transition from higher-order morphologies (vesicles) to lower-order morphologies (thermodynamically stable micelle structures) as a result of the self-adaptation behavior of the system was also proven.

### 6.3. Polymerization-Induced Electrostatic Self-Assembly

**Scheme 39:**
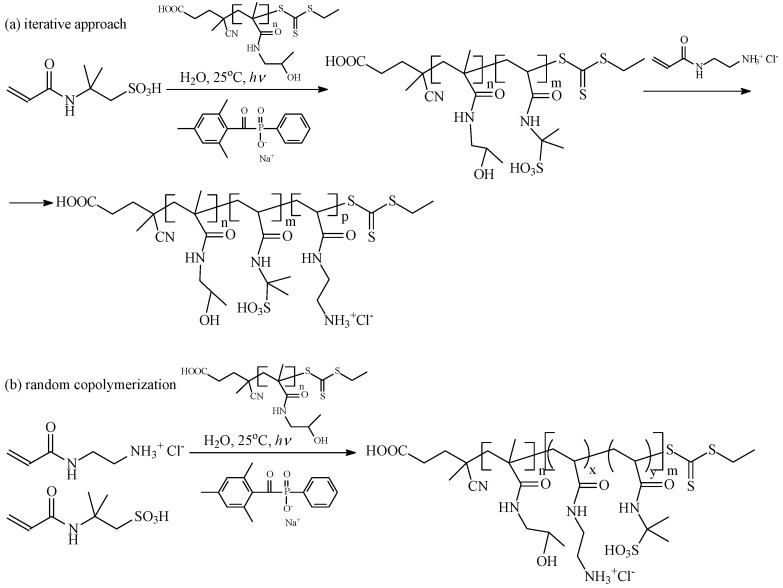
Scheme of iterative and random copolymerization of AMPS and AEAM in the presence of a nonionic macro-RAFT agent based on PHPMA [353].

The well-known ability of oppositely charged polyelectrolytes to form interpolyelectrolyte complexes was successfully applied in PISA techniques. In the first publication [353], the acceleration effect and selective monomer addition during RAFT copolymerization of the oppositely charged ionic monomers in a dilute aqueous solution at 25 °C were described. The reaction was performed using a non-ionic water-soluble macro-RAFT agent, poly(2-hydroxypropylmethacrylamide) (PHPMA), under visible light irradiation. When AMPS and AEAM were separately polymerized using PHPMA, AMPS was polymerized faster than AEAM. Polymerization in 1.0 M NaCl was faster than in water due to the weakening of the repulsion of ions. During iterative polymerization (Scheme 39a), a transparent gel was formed within 15 min or a milky gel in 60 min, suggesting the formation of water-insoluble PECs.

The electrostatic attraction promoted the enrichment of AEAM molecules into oppositely charged PHPMA-*b*-PAMPS coils in a dilute aqueous solution, thus accelerating the reaction. Random copolymerization of AEAM and AMPS (Scheme 39b) was characterized by selective monomer addition due to the ion-pairing of the oppositely charged monomers. Both monomers copolymerized at precisely the same rates at a 1:1 monomer ratio, although AMPS was more reactive than AEAM. Moreover, AEAM was polymerized faster than AMPS on decreasing molar [AEAM]/[AMPS] and more slowly on increasing, i.e., a faster reaction of the minor monomer component over the major one is achieved. The addition of NaCl suppresses the acceleration effect.

**Figure 9:**
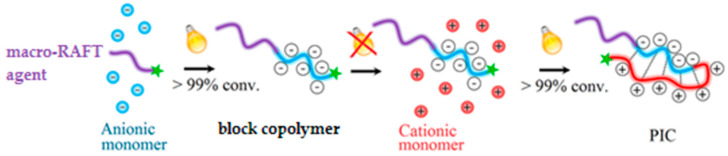
Scheme of a PIC-PISA process [354].

**Scheme 40:**
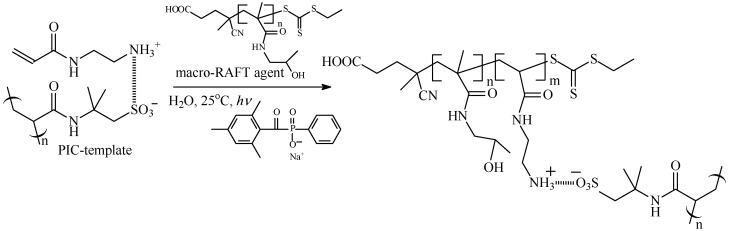
Scheme of aqueous PIC–PISA of AEAM polymerization using PAMPS-based PIC-template and nonionic macro-RAFT agent based on PHPMA [354].

A slightly changed approach based on polyion complexation (PIC) was suggested in [354]. When a polyelectrolyte and a polyion-neutral block copolymer of opposite charge are mixed, nanostructured polyion complexes (PICs) can be formed spontaneously in water [355]. Yu et al. called this approach PIC–PISA, implying that polyion complexation is the driving force of PISA (Figure 9). PIC–PISA was performed through dispersion polymerization of cationic monomer AEAM in water under visible light irradiation at 25 °C, using a nonionic macro-RAFT agent PHPMA in the presence of anionic PAMPS as a PIC-template (Scheme 40) [354]. A sphere-to-network transition occurred owing to the PIC of PAMPS with growing chains upon reaction close to the isoelectric point. Further reactions led to an increase in electrostatic repulsion, which promoted the split of networks and the rupture of spheres into fragments. Therefore, the free-flowing solution becomes a viscous liquid and a free-standing physical gel, and then it goes back into a viscous and free-flowing liquid. The structures of ionic PIC-template, growing chains, and nonionic macro-CTA, counterions, ionic strength, pH, and so forth are all important variables.

Later, this synthetic strategy was renamed polymerization-induced electrostatic self-assembly (PIESA) [356]. This strategy was validated by the sphere–colloidal branch/network–oppositely charged sphere–unimolecular polyion complex (uPIC) transition in photo-RAFT polymerization of an ionic monomer in the presence of polyion of opposite charge (1/2 mol. to monomer) in water at 25 °C. Using PIESA and the above-mentioned system, comprising AEMA, PAMPS, and PHPMA-based macro-RAFT agents, multi-compartmental vesicles and large-area ultrathin film can be achieved at 12–50% solids. It shows remarkable medium-tunable shape selectivity. Nanowire, dense-pore nanofilm can be prepared in a mixed methanol–water solvent.

The one-component PIC nanowire, film, vesicle, tube, and surface-charged vesicle have been prepared through PIESA using PAMPS, a PHPMA-based macro-RAFT agent, and histamine acrylamide hydrochloride (HisAM) or cystamine methacrylamide hydrochloride (CysMA) instead of AEMA [357,358]. PIESA showed sphere-film-vesicle transition and charge-/medium-dependent shape selectivity, and vesicle-polymerization has been fulfilled for the first time. Other living radical polymerizations are applicable to this robust room-temperature PIESA synthesis, especially Boyer’s PET-RAFT [359]. Thermal control of one-component low-dimensional PICs can be achieved using traditional thermal RAFT.

**Scheme 41:**
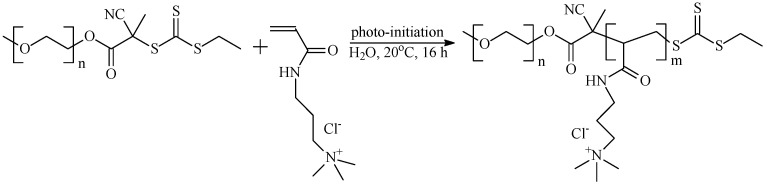
PIESA of 3-acrylamidopropyl trimethylammonium chloride mediated by PEG-terminated trithiocarbonate [325].

PIESA can be used to mediate the self-assembly behavior of short interfering RNA (siRNA, 19–20 bp) [325]. PEG-based macro-RAFT agent and siRNA taken in a molar ratio of 1:0.73 were used to provide block copolymerization of 3-acrylamidopropyl trimethylammonium chloride (APTAC) and simultaneous self-assembly with siRNA through the RAFT-PISA mechanism under the action of lithium phenyl-2,4,6-trimethylbenzoylphosphinate as the photo-initiator (Scheme 41). The electrostatic interaction between rigid siRNA and flexible polycations in PAPTAC chains drives the self-assembly, and the molecular rigidity of siRNAs leads to the directed self-assembly of siRNAs. When the PAPTAC chain is relatively short (degree of polymerization ~30), only a small amount of siRNA can be packaged in a random way, and non-spherical particles are formed. As the PATPAC block grows, more siRNA aggregates together and forms a lamellar structure. The positively charged PAPTAC chains can entangle around siRNA like ropes. The lamellar structure became large, soft, and bendable with the further growth of PATPAC. These self-assemblies are stable enough and provide effective encapsulation of siRNA, protecting it from RNase treatment.

**Scheme 42:**
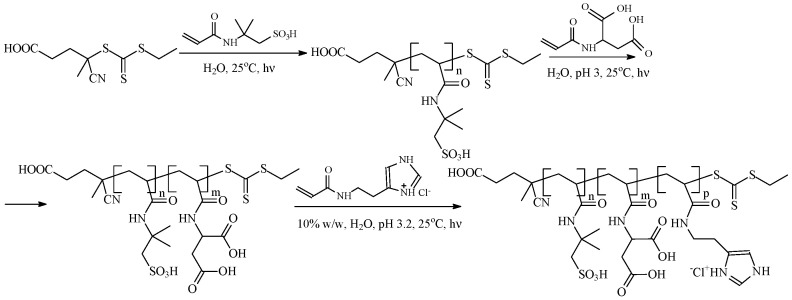
Scheme of like-charge PISA to produce PAMPS-*b*-PAspAm-*b*-PHisAm block copolymer dispersions [360].

The curious strategy to achieve electrostatic self-assembly was developed in [360] by a combination of seeded polymerization and electrostatic self-assembly. Luo et al. used the PAMPS macro-RAFT agent to perform PET-PISA of the L-aspartic acid acrylamide (AspAm) monomer. The final dispersion of ionic–nonionic block copolymer arising from spherical particles was used in a seeded polymerization of cationic HisAm monomer (Scheme 42) to produce particles with multilayer lamellar morphology.

### 6.4. Polymerization-Induced Hierarchical Self-Assembly

**Figure 10:**
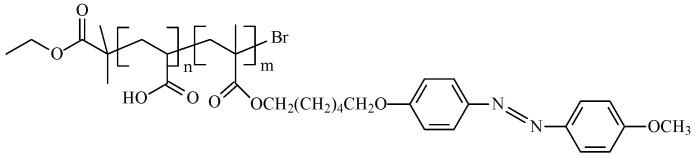
The structure of AB-diblock copolymer synthesis through ATRP of *t*-butyl acrylate and AzoMA [362].

Tunable anisotropic morphologies can be achieved by a combination of PISA and liquid crystalline (LC) ordering. This approach is known as polymerization-induced hierarchical self-assembly (PIHSA) and was first reported in [361]. The background of PIHSA was laid in [362,363]. Amphiphilic AB-diblock copolymers of azobenzene-containing polymethacrylate (PAzoMA) and PAA were synthesized through ATRP of *t*-butyl acrylate and AzoMA, followed by selective hydrolysis (Figure 10) [362]. This diblock copolymer has demonstrated the ability to form micelles in a selective solvent. The reversible trans-cis photoisomerization of azobenzene mesogens in PAzoMA occurred under alternating UV and visible light illumination, leading to reversible changes in micellar aggregates. UV-irradiation (trans-cis isomerization) disrupted micelles into single chains, while visible light (cis-trans isomerization) caused their reformation.

**Scheme 43:**
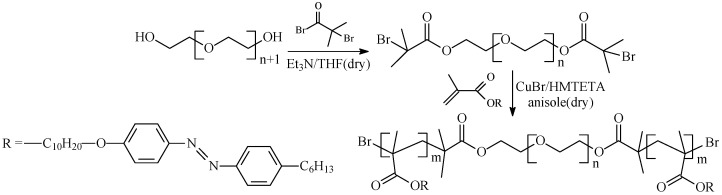
Scheme of ABA triblock copolymer synthesis through ATRP of *t*-butyl acrylate and AzoMA [363].

Similar behavior reveals an ABA-triblock copolymer comprising PAzoMA A-block and PEO B-block (Scheme 43) [363].

**Scheme 44:**
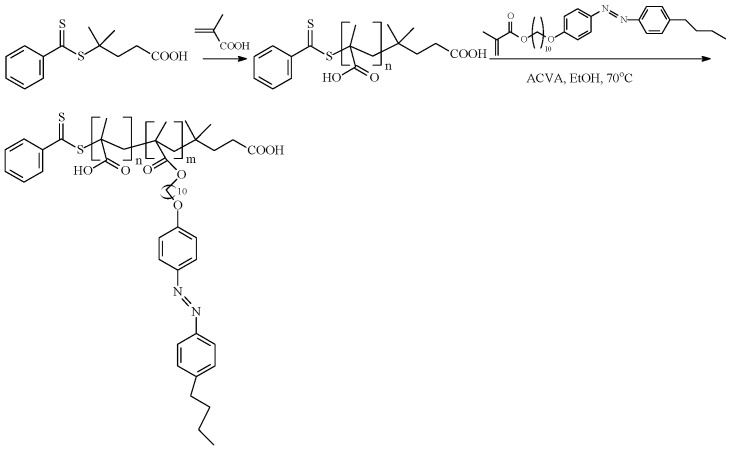
Dispersion PISA of AzoMA in ethanol using a PMAA-based macro-RAFT agent [361].

The morphological transitions are observed in thin films of triblock copolymers under the alternating action of UV and visible light. Based on these data, the dispersion polymerization of PAzoMA was performed in an ethanol solution in the presence of a PMAA-based macro-RAFT agent (Scheme 44) [361]. Guan et al. supposed that during PISA, the assembly of the block copolymers and the internal LC ordering in the core-forming block occur simultaneously. By tuning the volume fractions of the blocks, various morphologies could be obtained, including some unusual forms that have not been observed previously in PISA. The morphology changes from cuboids to 1D nanowires, then to lamellae, and finally to ellipsoidal vesicles with an increase in MW of PAzoMA block at a constant MW of PMAA block (approximately 112 units of MAA). A decrease in the length of the stabilizing block (38 units of MAA) results in ellipsoidal vesicles, lamellar, and worm-like morphologies, while an increase leads to the preferable formation of different worm-like morphologies.

**Figure 11:**
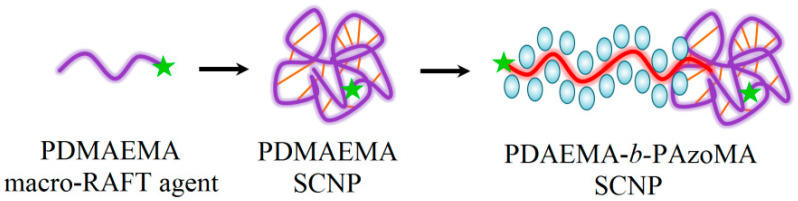
Schematic illustration of the formation of PDMAEMA(SCNP)-*b*-PAzoMA via RAFT dispersion polymerization [365].

Wen et al. have performed a number of studies to reveal the influence of polymerization conditions on the control of the morphology of nanoobjects formed in dispersion polymerization of AzoMA monomer. PDMAEMA with a dithiobenzoate end-group was used in [364] for dispersion PISA in butanol. Moreover, 1D morphologies were observed for a wide range of degrees of polymerization of both PDMAEMA and PAzoMA. The photoresponsive behavior of PDMAEMA-*b*-PAzoMA nanostructures was studied by alternate UV/visible light irradiation. UV irradiation leads to the transformation of worms and 1D nanowires into spheres or even vesicles, depending on the exposure time. These transformations are reversible and are observed during several cycles of irradiation. Single-chain nanoparticles (SCNPs) were synthesized by intra-chain cross-linking of a linear macro-RAFT agent based on PDMAEMA with 1,4-diiodobutane, followed by RAFT dispersion polymerization of AzoMA (Figure 11) [365]. The steric architecture of PDMAEMA SCNP stabilizers results in distinguishing self-assembly behavior. With the increasing degree of polymerization of linear PAzoMA blocks, spherical morphology transforms first into a cuboidal one, then into cuboids with holes, and finally into a hollow cuboid (holbrick-like morphology). The latter is maintained even after 24 months, indicating its thermodynamic stability.

The influence of stabilizing group Z in the initial RAFT agent on the PISA control and morphological transitions using PDMAEMA-*b*-PAzoMA is described in [366]. The morphological transformation from ribbons to twisted ribbons occurs in the poorly controlled PIHSA (for Z = SC_12_H_25_), while only wires and ribbons can be observed in the well-controlled PIHSA (for Z = Ph).

**Scheme 45:**
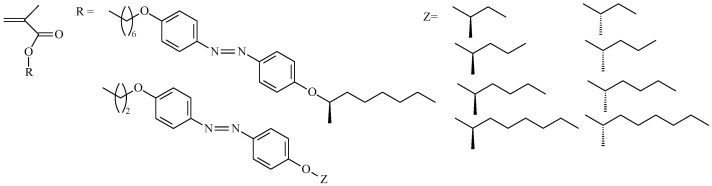
The chemical structure of chiral azo-containing methacrylates (AzoMA) used in PISA [367,368,369,370,371].

Similar studies of RAFT dispersion PISA of azo-containing methacrylate monomers with spacers containing 2–6 methylene groups were performed by the Znang group [367,368,369,370,371]. The main difference in the monomer structure is the presence of chiral tails in the para-position of the aromatic ring (Scheme 45). The particular structure of AzoMA monomers and associated morphological transitions of prepared block copolymers allowed authors to call this type of polymerization polymerization-induced chiral self-assembly (PICSA).

Thus, the dispersion polymerization of AzoMA monomer with six methylene groups in a spacer in the presence of a PMAA-based macro-RAFT agent results in the formation of chiral assemblies. The morphological transitions of spheres → worms → helical fibers → lamellae → vesicles → compound vesicles are achieved by tuning the degree of polymerization of PAzoMA core-forming blocks [368]. The decrease in the number of methylene groups to two leads to polymerization-induced chiroptical inversion [369]. The variation of the length of the chiral tail allows the formation of helical fibers [370]. Finally, the CO_2_-, photo-, and thermal-triple stimuli-responsive supramolecular chiral assemblies based on PDMAEMA as the solvophilic block and the chiral PAzoMA block as the core-forming block are obtained, which exhibit reversible morphology transitions (micelles → spheres → worms → vesicles) under the action of CO_2_, UV light, and temperature [371].

Semi-fluorinated methacrylates have shown themselves as versatile monomers for PISA [372]. Alcoholic dispersion polymerization of 2-(perfluorobutyl)ethyl methacrylate (FBEMA), 2-(perfluorohexyl)ethyl methacrylate (FHEMA), and 2-(perfluorooctyl)ethyl methacrylate (FOEMA) in the presence of a PDMAEMA-based macro-RAFT agent produces polymer assemblies with varying nanostructures depending on the side-chain lengths of the monomers. Block copolymers comprising PFBEMA can form spheres, worm-like micelles, and vesicles, while only spheres are generated by block copolymers with PFHEMA block. While PFOEMA has a liquid-crystalline nature, it can generate cylindrical micelles. Further interest in PDMAEMA-*b*-PFOEMA results in the development of programmable shape transformations of the synthesized nanoassembles due to taking advantage of the LC-to-isotropic phase transition [373]. The original ellipsoids were heated to 95 °C (above transition to isotropic phase) and then cooled in different manners, i.e., quenched by liquid nitrogen (ellipsoid → sphere), cooled to 15 °C with a cooling rate of 1 °C/min (ellipsoid), or quenched by 10 °C H_2_O (ellipsoid → faceted spheres). Spheres can be reconfigured to their original ellipsoidal shape by heating to 95 °C and cooling to 15 °C (1 °C/min). The polymerization temperature close to or far from the transition to the isotropic phase also allows tuning of morphology, resulting in ellipsoids at a polymerization temperature of 70 °C and cylindrical micelles at 50 °C.

Polymerization-induced crystallization-driven self-assembly may be considered a particular case of PIHSA. Hierarchical growth is controlled by polymerization, self-assembly, and crystallization that operate on different timescales, resulting in the formation of 1D, 2D, or 3D nanostructures. It was observed during ring-opening polymerization of L-lactide (LLA) in toluene in the presence of a PEG macroinitiator and the formation of PEG-*b*-PLLA [374]. No self-assembly is observed when the degree of polymerization of the PLLA block is below 20 units. The morphology depends on both the degree of polymerization of the PLLA block and the solid content. The non-equilibrium structures are formed due to fast polymerization relative to self-assembly and crystallization. However, later, the system relaxes to a lower energy state. For PLLA_45_-b-PEG_45_ (10% solids *w*/*w*), the PLLA crystallinity increased rapidly during the first three hours to 51% and then increased slowly to ~81% around 24 h, provoking self-assembly. With an increase in the degree of polymerization of the PLLA block, the initial rate of crystallization increases.

**Figure 12:**
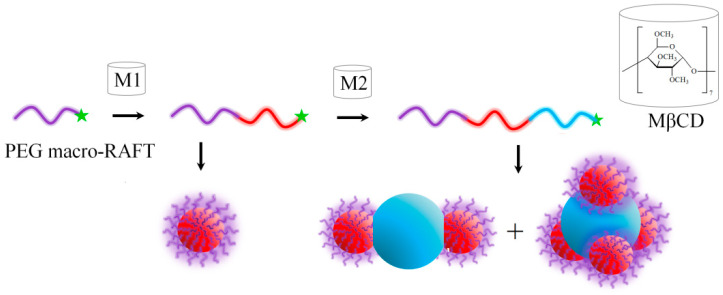
Scheme of the synthesis of CMs using MβCD/monomer complex via PIHSA [375].

The unusual approach to PIHSA aiming to produce a colloidal molecule (CM) comprising ABC triblock copolymers is suggested in [375]. In this case, the selection of monomers plays a key role in achieving hierarchical self-assembly. Physical compatibility, interfacial energy, glass-transition temperature, and composition order of the blocks determine the desired morphology. Block A is a stabilizing block, and it is hydrophilic if self-assembly occurs in aqueous solutions. To access CM topology, phase separation between blocks B and C is required, while the interfacial tension between block B and aqueous solution should be higher than that between block C and solution. If the latter condition is not met, then raspberry-like micelles are likely to form [376]. The glass transition temperature of core-forming blocks impacts the chain mobility and the morphology transitions [203]. To perform aqueous RART dispersion polymerization of water insoluble monomers, the methylated-β-cyclodextrin (MβCD) was complexed with a hydrophobic monomer (styrene, *tert*-butyl acrylate (tBA) or *tert*-butyl methacrylate (tBMA)) to make a homogeneous aqueous solution by “host–guest” inclusion and polymerized using PEG-based macro-RAFT agent (block A) (Figure 12). Depending on the monomer order, various morphologies are obtained. Thus, PEG-*b*-PS-*b*-PtBA forms CMs, PEG-*b*-PtBA-*b*-PS forms core–shell–corona micelles, and PEG-*b*-PtBMA-*b*-PS forms raspberry-like micelles.

**Scheme 46:**
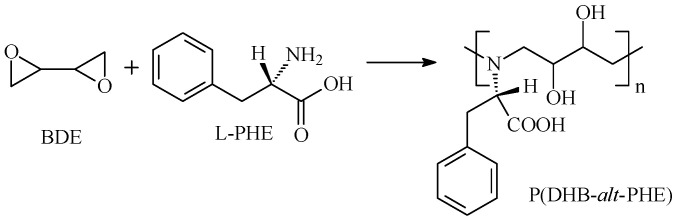
Formation of P(DHB-*alt*-PHE) through PISA [374].

Combination of liquid–liquid phase separation with PISA results in the one-pot and scalable preparation of hierarchical bishell capsules from nanosheets with ultrathin lamellae phase (sub-5 nm), micro-flakes (multiple nanosheets), and unishell capsules to final bishell capsules in a bottom-up sequence [377]. L-phenylalanine (L-PHE) aqueous solution spontaneously generates coacervate with the addition of potassium carbonate. The further addition of hydrophilic 1,3-butadiene diepoxide (BDE) causes the epoxy-amine click polymerization at the surface of the coacervates to form the alternating poly(dihydroxy butylene-*alt*-2-tert-amine-3-phenylpropanoic acid) P(DHB-*alt*-PHE) (Scheme 46). The latter undergoes self-assembly to form an ultrathin lamella phase. The lamellae grow into thin sheets and form micro-flakes. Finally, the flakes form the unishell capsule. Afterward, click polymerization goes on through the cavities on the surface of the unishell capsule, and an upper second shell consisting of the newly formed micro-flakes is formed, resulting in the formation of bishell capsules. Xu et al. called this approach coacervate-assisted polymerization-induced self-assembly; however, it is a typical example of PIHSA.

The combination of two driving forces, namely hierarchical and electrostatic self-assembly, is reported in [378]. This formulation contains the cationic monomer HisAM, the PHPMA-based macro-RAFT agent, and the anionic block copolymer micelles PAMPS-*b*-PDAAm. RAFT polymerization yields a cationic diblock copolymer, PHisAM-*b*-PHPMA, that can co-assemble with the anionic micelles to form. The incompatibility of non-ionic PDAAm blocks and growing PIC causes microphase separation and the formation of mutually incompatible domains. Depending on the polymerization conditions, the multicompartment micelles, their colloidal nanosheets, and nanocages can be obtained.

### 6.5. Polymerization-Induced Particle or Surface Self-Assembly

**Figure 13:**
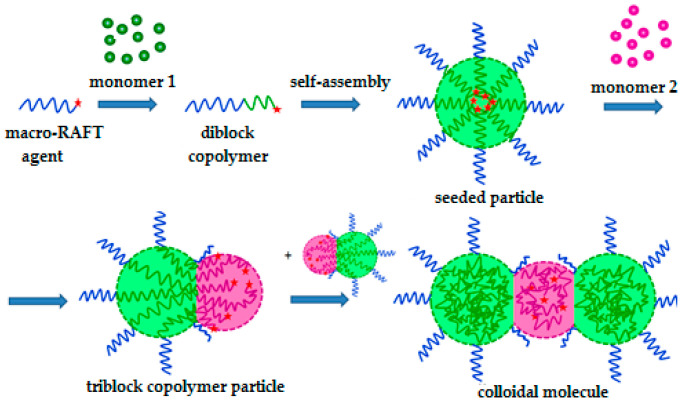
The synthesis of AB2-type colloidal molecules through PIPA [376].

Colloidal molecules are well-defined colloidal clusters with precise symmetry of molecular structures and have drawn extensive interest due to their unique properties and potential in biological and medical applications [379]. The synthesis of well-defined colloidal molecules can be achieved directly via seeded polymerization without the preparation and purification of patchy intermediates (Figure 13) [376]. This strategy is known as polymerization-induced particle assembly (PIPA). Firstly, the PIPA strategy was applied to the seeded polymerization of HPMA in the presence of spherical seeds of diblock copolymers of PDMAEMA-*b*-PBzMA. PHPMA blocks of triblock copolymers PDMAEMA-*b*-PBzMA-*b*-PHPMA connect triblock copolymer particles into AB_2_-type colloidal molecules directly during polymerization. Depending on the length of the third block in the growing ABC triblock copolymer, colloidal molecules may have a symmetric spherical morphology (the C-block is short). After reaching the critical length of the C-block, the Janus micelles are able to form AB_n_-type colloids. The PIPA mechanism includes the following: (1) the formation of spherical core–shell seeds of PDMAEMA-*b*-PBzMA; (2) the formation of a triblock copolymer with PHPMA blocks, which phase separates with PBzMA blocks and aggregates into PHPMA micro-domains; (3) particle coalescence after PHPMA domains reach a critical size and the formation of colloidal clusters. The structure of colloidal clusters is determined by the volume ratio V_PHPMA_/V_PBzMA_. ABn-type colloidal molecules are formed when V_PHPMA_/V_PBzMA_ > 1.

This strategy was applied to the synthesis of CH_4_-like colloidal tetrahedra based on PDMAEMA-*b*-PBzMA-*b*-PDAAm and PDMAEMA-*b*-P(2-(perfluorobutyl)ethyl methacrylate)-*b*-PBzMA [380].

**Figure 14:**
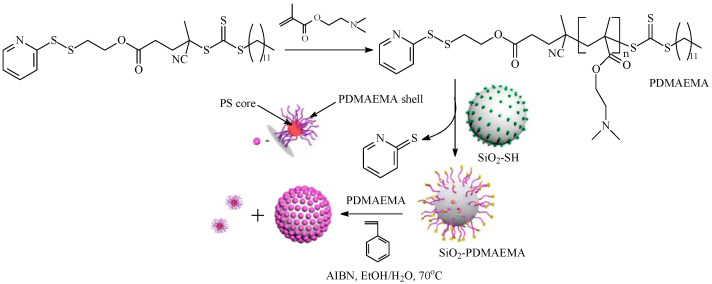
Scheme of PISSA and formation of silica particles with grafted micelles of PDMAEMA-*b*-PS [381].

The fabrication of hierarchical surface nanostructures becomes possible with the polymerization-induced surface self-assembly (PISSA) approach [381]. In this approach, two macro-RAFT agents are used in dispersion polymerization. One of them is grafted on silica particles, and the other is molecularly dissolved in solution. It results in the formation of surface micelles with different morphologies and sizes on silica particles. The morphology of surface micelles depends on the monomer conversion and the degree of polymerization of the grafted macro-RAFT agent. A morphological transition from spherical s-micelles to layered structures is observed in macro-RAFT agents with low MW and an increase in monomer conversion. The growth of the MW of macro-RAFT agents leads to a rise in the average size of micelles. According to this approach (Figure 14), the surface of SiO_2_ particles was modified to produce thiol-functionalized silica particles, which were subjected to a thiol–disulfide exchange reaction with a PDMAEMA-based macro-RAFT agent. The silica particles with grafted PDMAEMA chains were used as a seed in the dispersion polymerization of styrene. The addition of a free PDMAEMA macro-RAFT agent results in the formation of both surface micelles and free micelles.

PISSA can be used to fabricate a biosurface [381]. This term means a surface decorated with biological objects such as enzymes, polysaccharides, DNAs, or cells. An interesting example of biosurface formation is presented in [382]. In this case, RAFT dispersion polymerization of NIPAM is used. The surface of silica particles is modified with a small-molecule RAFT agent using a thiol–disulfide exchange reaction. Bovine serum albumin (BSA) is modified with the same RAFT agent according to a similar procedure. Thus, grafted and free RAFT agents are used simultaneously, resulting in the formation of PNIPA layers on the surfaces of silica particles with BSA on top of the particles. At a temperature below the LCST of PNIPA, the activity of the immobilized BSA is retained, and conversely, the BSA activity decreases above the LCST due to the hydrophobic interaction between PNIPA and BSA.

Later, PISSA was applied to prepare Janus surface micelles on the surface of silica particles, which were used for enzyme immobilization [383]. PDMAEMA macro-RAFT agent is grafted to silica particles, while poly(di(ethylene glycol) methyl ether methacrylate) (PDEGMA) is prepared by solution polymerization. Both grafted and free macro-RAFT agents are used in RAFT dispersion polymerization of styrene to synthesize Janus surface micelles with PS cores and PDMAEMA/PDEGMA shells. On one side of a micelle, PDMAEMA brushes connect the micelle and the silica particle. On the other side, PDEGMA chains are stretched away from the PS core into the medium. The further quaternization of PDMAEMA allows the immobilization of enzymes through electrostatic interaction.

Asymmetric colloidal particles can be fabricated through a similar approach using silica particles decorated with RAFT agents and PEG-based macro-RAFT agents in styrene dispersion polymerization [384]. Surprisingly, a polymer nodule appears on each SiO_2_ particle with an increase in monomer conversion. The addition of a free small-molecule RAFT agent allows the synthesis of morphology-tunable asymmetric hybrid particles.

## 7. Computer Simulation Studies of PISA

As it is seen from the chapters presented above, there is a whole spectrum of parameters that have a crucial influence on the morphology of self-assembled structures in PISA-regulated processes. Among them are the choice of monomers and macro-agents, solvent, initiator, as well as the relative concentration of reacting components, temperature, and polymerization technique. Clearly, the experimental study of the multiple parameter-dependent phase diagrams of such systems is time-consuming and requires a lot of effort and resources. This problem can be solved by using in silico investigations. Computer simulation methods, such as dissipative particle dynamics (DPD [385,386]), help to gain insight and perform an in-depth analysis of complicated polymer systems’ behavior, which was previously shown, for example, for block copolymer self-assembly research [387,388,389,390,391,392,393].

Computer simulations of PISA mechanisms have begun relatively recently, with the growth of methodology and computational resources. One of the first studies on this topic was published in 2016 by Huang et al. [394]. In this work, only chain propagation is considered, while chain transfer and chain termination are omitted. Thus, these simulations may be referred to as living anionic PISAs. Huang et al. compare the solution polymerization and PISA results resulting in the formation of an AB-diblock copolymer (the degree of polymerization of A-block is 3; the targeted degree of polymerization of B-block is 7). According to DPD simulations, the initial kinetics of PISA and solution polymerization are similar and follow the first-order rule. However, with time, PISA kinetics derives from the first-order rule and becomes slower in contrast to solution polymerization kinetics. The simulations of particles’ morphology were performed for the repulsive parameter between hydrophilic A-block and hydrophobic B-block equal to 50 and that between hydrophobic B-block and solvent molecules equal to 75. The formation of small aggregates occurred for diblock copolymer A_3_B_2.2_, followed by a transition to worm-like micelles for diblock copolymer A_3_B_3.3_. As the polymerization continues, the worm-like micelles gradually transform into vesicles (A_3_B_5.7_). Finally, a large-compound micelle formed for the diblock copolymer A_3_B_6.9_. It is difficult to compare the results of the simulations with those from the experiment since, in practice, such experimental conditions are not used. A similar approach was then used to model PISA in polymers of more complicated morphology, such as star [395] and rod-coil [396] block-copolymers.

In a paper by Lv et al. [397], the DPD method was used to explore the self-assembly behavior of side-chain liquid crystalline copolymers. It was shown that such copolymers can self-organize in micelles, vesicles, and deformed structures as rectangle cylinders, depending on the concentration of copolymers and the length of the coil blocks. Simulation reproduces the phenomena found in the experimental works [398]. For block copolymers PEG-*b*-PAChol (polyacrylate containing a cholesteryl-based mesogen side group), the formation of vesicles was observed when the ratio of the degrees of polymerization of PEG and PAChol blocks exceeded ~3 [398]. When this ratio was less than 2, spherical micelles were formed. The transformation from vesicles to spheres with increasing the number of side chains correlates with simulation results [397]. The appearance of longer rod-like micelles with an increase in the number of side chains in the experiment qualitatively accords with the simulated transition from vesicles to cylinders as the number of side chains increases.

The variation of PISA, PICA, was modeled in [349].

In an attempt to develop a realistic simulation strategy for PISA formation of PAA-*b*-PS in methanol through the RAFT technique, Yan et al. [399] proposed a model for the dynamic PISA process based on input simulation parameters obtained from the experimental data and studied a set of polymerization rate values. It was demonstrated that morphologies are affected by the polymerization rate. Only spherical micelles are found in PISA, which has a fast polymerization rate. In contrast, fusion between micelles is observed for PISA with a slow polymerization rate. In another work [400], a precise mesoscopic DPD model of the PISA of P4VP-*b*-PS was presented. The dynamics of PISA under specific solvent and monomer conditions and different polymerization rates were investigated to help the researchers optimize the PISA strategy for the formation of targeted morphologies. The influence of surface compatibility with the monomer species on the polymerization-induced phase separation is explored in [401] both theoretically and experimentally.

Gavrilov et al. [36] have used the DPD method to compare the phase diagrams for the solutions of pre-synthesized ideal (monodisperse) AB-diblock copolymers and diblock copolymers formed during PISA and to distinguish how the peculiarities of the polymerization process influence the system properties. In both cases, the degree of polymerization of the A-block was taken as 6, while the degree of polymerization of the B-block varied from 12 to 42. The polymer—solvent (S) interaction parameter χ was taken as χ_AS_ = 0 (for A-block) and χ_BS_ = 1.9 (for B-block); χ_AB_ = 1.9. Termination and transfer reactions were neglected, as in [394]. As the degree of polymerization (DP) of the solvophobic B-block increases, the transitions occur in the following order: spherical micelles → cylinders → vesicles, in accordance with theoretical predictions. A rather wide transition region between the spherical and cylindrical morphology was found (DP_B_/DP_A_ = 3–4, at polymer volume fraction 0.05), in which the system contains a mixture of spheres and short cylinders, which appear to be in dynamic equilibrium. Upon increasing the polymer concentration in the system (at polymer volume fraction 0.3), the transition region between the spheres and cylinders shifts towards lower values (DP_B_/DP_A_ = 2.5–3.7). For PISA, the transitions occur at a larger B-block length. The shift of the transition position between the spheres and cylinders is small (ΔDP_B_/DP_A_ < 0.05), while the transition between the cylinders and vesicles is rather sharp (ΔDP_B_/DP_A_ > 0.25). Gavrilov et al. proposed that the reason for such behavior is the dispersity of the core-forming blocks (Đ = 1.03 for cylinders and Đ = 1.09 for vesicles): the presence of the short and long blocks being located at the micelle interface and in its center, respectively, helps to reduce the entropy losses due to the insoluble block stretching, which leads to the increased stability of more curved micelles. In real PISA protocols, the dispersity of block copolymers (both core-forming and shell-forming blocks) is higher. Thus, the co-existence of spheres and worms, worms, and vesicles observed in the experiment (see Figure 4 and Figure 5) may be caused by the dispersity of block copolymers, as shown by Gavrilov et al. These results raised an important question about the necessity of thorough simulation and investigation of the whole synthetic route, including all possible side reactions as well as the presence of dormant chains, in order to better understand and predict the experimental data.

In the above-mentioned studies, the simplest polymerization scheme was employed, meaning that only initiation and propagation reactions were considered without chain activation–deactivation and termination reactions accounting, thus mimicking an ideal living polymerization. Such omissions led to the diminishment of the polydispersity of the forming chains and the absence of more complicated chain morphologies, such as triblock forming through recombination mechanisms. However, these factors can significantly influence the phase diagrams obtained in laboratory experiments. The first work with a realistic polymerization scheme resembling the real process of RDRP for the simulation of ATR-PISA appeared in 2022, written by Petrov et al. [402]. The influence of the reversible activation–deactivation and the termination via recombination reactions on the phase diagrams was evaluated. Similar initial parameters were used for simulation, as in [36]. As it was predicted in [36], the termination reactions had a strong impact on the regions of cylindrical micelles and vesicle formation. The shift of the transition position between the spheres and cylinders is about ΔDP_B_/DP_A_ ~0.5 for 95% of terminated chains, while the transition between cylinders and vesicles is about ΔDP_B_/DP_A_ ~0.5 for 50% of terminated chains and more than 1.0 for 95% of terminated chains. The DPD model of RAFT polymerization, which considers the main reactions of the experimental RAFT process, was proposed by Gavrilov [403], showing, among other results, that in RAFT PISA, the incompatibility between the RAFT end group and other species has a significant impact on the forming morphologies. The work [404] provides a neat comparison between the experimental PISA of poly(cholesteroyl methacrylate)-*b*-PS and simulations.

Studies utilizing dissipative particle dynamics simulation methodology occupy the vast majority of the theoretical investigation of PISA, yet other approaches also enrich this field. The molecular dynamics simulation method was used in [405] to show the peculiarities of cluster aggregation mechanisms appearing during PISA in emulsions. The findings can explain the change in morphology from spheres to fibers and vesicles depending on the polymer architecture. In a very useful study for the experimentalists, the group of O’Reilly reported an in silico method for the prediction of monomers suitable for PISA by evaluating the change in solvophobicity of the growing polymer chain during polymerization and, thus, its ability to form distinct morphologies [406]. As proof, five new monomers were found to be suitable for use in aqueous PISA via reversible addition–fragmentation chain transfer (RAFT) polymerization, which was confirmed by laboratory experiments. In a very recent work by Lu [349], the first machine learning framework for data-driven prediction of morphological outcomes and recommendation of experimental designs in PISA was presented. The model showed reasonable performance in predicting the forming morphologies using the inputs of monomers, polymers, and reaction condition descriptions. The proposed method in the future can drastically reduce the number of practical experiments required to obtain the self-assembled structures of the desired shape, yet its performance is limited by the current training dataset based on available experimental results.

To summarize, under the insightful guidance of computer simulation, the PISA investigations are expected to achieve new breakthroughs.

## 8. Outlook and Perspectives

Summarizing, PISA has enriched conventional emulsion and dispersion polymerizations with the ability to prepare nano-objects with different morphologies at relatively high polymer concentrations. It may be performed in various media, over a wide range of temperatures, and using different polymerization mechanisms [170,201,202,203,204,206,407,408]. The key factors controlling the typical particle morphologies (spheres, worms, and vesicles) in PISA processes are the degree of polymerization of core-forming and shell-forming blocks and the content of solids. However, new techniques have emerged for constructing more complex morphologies of AB- and ABC-block copolymer particles. These include thermo-responsive properties of core-forming blocks (PITSA), simultaneous growth of block copolymers and homopolymers (PICA), formation of interpolyelectrolyte complexes by oppositely charged polyelectrolytes (PIESA), ordered structures formed by liquid crystal polymers (PIHSA), use of seeded particles (PIPA), and modified surfaces (PISSA). Additionally, degradable PISA nanoparticles can be produced, which may include shell-degradable blocks composed of peptide-, protein-, DNA-, or polysaccharide-stabilizing blocks or core-degradable hetero-chain blocks typically obtained through ring-opening polymerization [408].

Some dispersion PISA formulations performed in non-polar media offer potential industrial applications. Among them, poly(2-ethylhexyl acrylate)-*b*-poly(methyl acrylate) spherical nanoparticles prepared in isododecane may be useful for certain cosmetic formulations [409,410,411]. Dispersions of poly(stearyl methacrylate)-*b*-PBzMA-*b*-PEGDMA spherical particles with cross-linked cores are potentially useful as lubricant additives for automotive engine oils [412]. Dispersions of block-random copolymers, such as P(lauryl methacrylate-*co*-glycidyl methacrylate)-*b*-PMMA, cause a reduction in the friction coefficient after treatment of stainless steel due to chemical adsorption via ring-opening of the epoxy groups by reaction with the Fe–OH groups of the steel [413]. Dispersion of block copolymer particles prepared in non-polar media may be successfully used for the formation of water-in-oil (w/o) Pickering emulsions [414,415].

Aqueous dispersion and emulsion PISAs are of great interest due to their green chemical processes and high efficiency in producing a wide range of morphologies [204]. These formulations are particularly appealing for drug delivery and other biomedical applications [170]. For example, biocompatible thermo-responsive poly(glycerol monomethacrylate)-*b*-PHPMA particles can undergo a reversible worm-to-sphere transition upon cooling. This reversible gelation-degelation process, where a soft, free-standing physical hydrogel transforms into a low-viscosity dispersion, provides a highly convenient route to sterilizable gels [416]. The successful loading of guest compounds during PISA enables the loading of drug molecules as well [417,418]. O‘Reilly et al. demonstrated efficient loading of L-asparaginase into vesicles. After encapsulation, the morphology of the vesicles remained intact, and the encapsulated enzyme exhibited higher proteolytic stability than the free enzyme in vitro and in vivo [419]. Prodrug-core cross-linked nanoparticles with enhanced structural stability, containing camptothecin linked to the polymer chains via labile disulfide bonds, showed excellent anticancer efficiency against HeLa cells [420]. Post-modification of PISA-generated nanoparticles for therapeutic applications has also been implemented [421]. The dispersion of block copolymer particles with vesicular morphology has drawn interest as mimicking systems for living cells [422,423] and enzymatic nanoreactors [424]. Other applications of PISA include cellular imaging [425,426], catalysis using ligand-containing polymer nanoparticles [427,428], and using nanogels to stabilize and support Pd nanoparticles [429].

The commercialization of PISA protocols requires the improvement of PISA throughput. Significant progress has already been made in this area. PISA synthesis can be carried out under a nitrogen atmosphere using an automated synthesizer unit [430]. Oxygen inhibition can be minimized by performing chemical deoxygenation of the reaction mixtures using either enzymes or photochemistry [431,432,433]. PISA can be carried out at microliter (or even smaller) scales [434]. Oxygen-tolerant photo-initiation-enabled PISA synthesis allows for direct synthesis in 96-well microliter plates, facilitating the convenient parallel production of self-assembling nanoparticles on a laboratory benchtop [435]. The PISA process can also be conducted under continuous flow conditions using a stainless-steel tubular reactor through thermally initiated RAFT emulsion polymerization [436]. A continuous-flow PISA process under photo-initiating conditions is also possible [437,438]. All these approaches have significant promise for the successful implementation of PISA on a commercially viable scale.

Thus, future industrial applications of PISA should be preferably dealt with aqueous formulations and non-thermal initiation methods, which provide oxygen-tolerant PISA in open vessels, as well as continuous-flow methods of PISA, which can provide a possible route to mass production.

## 9. Conclusions

In this review, we have considered the recent achievements of PISA in producing gradient and block copolymer particles with a diverse set of morphologies. During the last 5 years, PISA has made a great leap forward in producing novel polymeric morphologies, and such swift development has fair origins. Firstly, PISA abandoned the conception of a nonselective solvent as a necessary prerequisite for the synthesis of AB- and ABC-block copolymers. PISA may work in any solvent in which at least one block among the three is soluble. Such an approach greatly expands the number of combinations of monomers suitable for copolymerization, and such diversity is unreachable for other techniques. Secondly, PISA, based on “living” or RDRP reaction mechanisms, enables sequential morphological transformations of polymer particles with time upon the gradual growth of insoluble blocks. It means that time becomes a powerful tool for morphological regulation. By terminating the reaction at a chosen moment in time, one can get polymer particles with the desired morphology. Thirdly, PISA proposes great facilities for the regulation of core–shell–corona surface tension through the synthesis of gradient copolymers. Such regulation is a key point in the regulation of the morphology of polymer particles. We believe that the abovementioned set of novel facilities, proposed by PISA, will very soon make this method the chief technique for the synthesis of block-copolymer nanoparticles with desired morphologies.

The current drawbacks of PISA stem directly from the disadvantages of the polymerization mechanisms. For example, the value of achievable molecular masses rarely exceeds 20–40 kDa in the RDRP techniques, and the researchers need to keep a balance between good control over MWD and high MWs. Nevertheless, the synthetic methods will continue to improve, thus making PISA more powerful in facilities and more comfortable to use with time.

The proposed review demonstrates a great deal of accumulated experimental data concerning the relationship between the chemical nature and composition of copolymers, the mechanisms of their synthesis by PISA, and the acquired morphology. We believe that it could be a good starting point for theoretical generalizations concerning the morphological transitions of ABC-triblock copolymers in selective solvents. We hope that our review may serve as one of the motives for the formulation of such a challenging task as well as a good reference during the quest for its resolution.

## Data Availability

Not applicable.

## Abbreviations

2VP	2-vinylpyridine
4VP	4-vinylpyridine
AA	acrylic acid
AEAM	2-aminoethylacrylamide hydrochloride
AIBN	2,2′-azobis(isobutyronitrile)
AMPS	2-acrylamido-2-methylpropanesulfonic acid
ANa	sodium acrylate
APTAC	acrylamidopropyl trimethylammonium chloride
AspAm	L-aspartic acid acrylamide
ATRP	atom transfer radical polymerization
ATR-PISA	atom transfer radical polymerization-induced self-assembly
AzoMA	azobenzene-containing methacrylate
BA	*n*-butyl acrylate
BDE	1,3-butadiene diepoxide
BIS	N,N’-methylenebisacrylamide
BNBE	2,3-bis(2-bromoisobutyryloxymethyl)-5-norbornene
BSA	bovine serum albumin
BzMA	benzyl methacrylate
CM	colloidal molecule
CMC	critical micelle concentration
CMRP	cobalt-mediated radical polymerization
CysMA	cystamine methacrylamide hydrochloride
DAAm	diacetone acrylamide
DEA	N,N-diethylacrylamide
DEGA	di(ethylene glycol) methyl ether acrylate
DEGMA	di(ethylene glycol) methyl ether methacrylate
DMA	dimethylacrylamide
DMAEMA	2-(dimethylamino)ethyl methacrylate
DMAP	4-dimethylaminopyridine
DMF	dimethylformamide
DP	degree of polymerization
DPD	dissipative particle dynamics
EA	(2-ethylhexyl)acrylate
EG	ethylene glycol
EGDMA	ethylene glycol dimethacrylate
EO	ethylene oxide
FBEMA	2-(perfluorobutyl)ethyl methacrylate
FHEMA	2-(perfluorohexyl)ethyl methacrylate
FOEMA	(perfluorooctyl)ethyl methacrylate
HFBA	2,2,3,4,4,4-hexafluorobutyl acrylate
HisAM	histamine acrylamide hydrochloride
HPMA	2-hydroxypropyl methacrylate
I	isoprene
ICAR	initiators for continuous activator regeneration
LC	liquid crystal, liquid crystalline
LCST	lower critical solution temperature
LAP	living anionic polymerization
LA-PICA	living anionic polymerization-induced cooperative assembly
LA-PISA	living anionic polymerization-induced self-assembly
LPHE	L-phenylalanine
LVC	large compound vesicles
MAA	methacrylic acid
MβCD	methylated-β-cyclodextrin
MMA	methyl methacrylate
M-TEMPO	4-methoxy-2,2,6,6-tetramethylpiperidine1-oxyl
MW	molecular weight
MWD	molecular weight distribution
NCA	α-amino acid N-carboxyanhydride
NCA-PISA	α-amino acid N-carboxyanhydride polymerization-induced self-assembly
NIPA	N-isopropylacrylamide
NMP	nitroxide-mediated polymerization
NM-PISA	nitroxide-mediated polymerization-induced self-assembly
*NVP*	*N*-vinylpyrrolidone
OEGA	oligo(ethylene glycol) methyl ether acrylates
OEGMA	oligo(ethylene glycol) methyl ether methacrylate
OFPA	2,2,3,3,4,4,5,5-octafluoropentyl acrylate
OMRP	organotellurium-mediated radical polymerization
ONBDM	7-oxanorborn-5-ene-exo-exo-2,3-dicarboxylic acid dimethyl ester
P	poly when followed by monomer abbreviation, i.e., PAA is equal to polyacrylic acid
P_A_, P_B_	degrees of polymerization of blocks A and B
PET	photoinduced electron/energy transfer
PIC	polyion complexation, polyion complex
PICA	polymerization-induced cooperative assembly
PICSA	Polymerization-induced chiral self-assembly
PIESA	polymerization-induced electrostatic self-assembly
PIHSA	polymerization-induced hierarchical self-assembly
PITSA	polymerization-induced thermal self-assembly
PIPA	polymerization-induced particle-assembly
PISSA	polymerization-induced surface self-assembly
PISA	Polymerization-induced self-assembly
PMDETA	N,N,N′,N″,N″-pentamethyldiethylenetriamine
PRIMSA	polymerization-reaction-induced molecular self-assembling
RAFT	reversible addition–fragmentation chain transfer
RAFT PISA	reversible addition–fragmentation chain transfer polymerization-induced self-assembly
RDRP	reversible deactivation of radical polymerization
ROMP	ring-opening metathesis polymerization
ROM-PISA	ring-opening metathesis polymerization-induced self-assembly
ROP	ring-opening polymerizations
S	styrene
SCNP	single-chain nanoparticle
siRNA	short interfering RNA
SG1	N-*tert*-butyl-N-(1-diethyl phosphono-2,2-dimethylpropyl) nitroxide
SSNa	sodium styrene sulfonate
*t*BA	*tret*-butyl acrylate
*t*BMA	*tret*-butyl methacrylate
TeMe	methyltellanyl
TEMPO	(2,2,6,6-tetramethylpiperidin-1-yl)oxyl
THF	tetrahydrofuran
TMEDA	N,N,N′,N′-tetramethylethylenediamine
uPIC	unimolecular polyion complex
UCST	upper critical solution temperature
V-50	2,2′-azobis(2-methylpropionamidine) dihydrochloride azo initiator
VEA	N-(4-vinylbenzyl)-N,N-diethylamine)
ZnTMPyP	zinc meso-tetra(N-methyl-4-pyridyl) porphyrin tetrachloride
